# Broad systemic T cell immunity to *Salmonella enterica* serovar Typhi in U.S. healthy adults

**DOI:** 10.3389/fimmu.2026.1940148

**Published:** 2026-10-01

**Authors:** Laura E. Carreto-Binaghi, Jayaum S. Booth, Rezwanul Wahid, Rosangela Salerno-Goncalves, Franklin R. Toapanta, Marcelo B. Sztein

**Affiliations:** 1 Center for Vaccine Development and Global Health, University of Maryland School of Medicine, Baltimore, MD, United States; 2 Department of Pediatrics, University of Maryland School of Medicine, Baltimore, MD, United States; 3 Department of Medicine, University of Maryland School of Medicine, Baltimore, MD, United States; 4 Tumor Immunology and Immunotherapy Program, University of Maryland Marlene and Stewart Greenebaum Comprehensive Cancer Center, Baltimore, MD, United States

**Keywords:** baseline immunity, high dimensional data analysis, mass cytometry, multifunctional responses, *S.* Typhi, *Salmonella enterica* serovar Typhi, T cell-mediated immunity

## Abstract

**Introduction:**

T cell mediated immunity (T-CMI) is an important component of effective host immunity against intracellular pathogens such as *Salmonella enterica* serovar Typhi (*S.* Typhi; ST). Baseline T-CMI responsiveness to *S.* Typhi has been previously described in healthy individuals, likely reflecting cross-reactive responsiveness to other *Enterobacteriaceae* within the gut microbiota. Interestingly, T cell baseline responsiveness has been associated with protection or susceptibility in a human challenge model following exposure to wild-type (wt) ST. To better understand the contribution of this baseline responsiveness to immunity against ST infection, an in-depth characterization of T cell immunity is needed.

**Methods:**

Here, peripheral blood mononuclear cells from eighteen healthy volunteers with no history of typhoid fever vaccination or infection were co-cultured with autologous B-lymphoblastoid cell lines (B-LCL) previously infected with wt ST as antigen presenting cells. Non-infected B-LCL (NI) were used as controls. After 18 h or 40 h of stimulation, cells were stained with a 52-parameter mass cytometry antibody panel and analyzed using manual gating, dimensionality reduction and unsupervised clustering approaches. We studied conventional CD4^+^ and CD8^+^ T memory cells, including T effector memory (T_EM_), T_EM_ re-expressing CD45RA (T_EMRA_), and T central memory (T_CM_), CD4^+^ and CD8^+^ T stem cell memory (T_SCM_), CD4^+^ circulating T follicular helper (cT_FH_), CD4^+^ T regulatory (T_reg_), and CD8^+^ T regulatory precursors (T_rp_), as well as non-conventional CD8^+^ mucosal associated invariant T (MAIT), T gamma delta (Tγδ), and Natural-Killer T (NKT) cells.

**Results:**

All studied cell subsets showed significant (p<0.01) baseline responsiveness to ST-infected target cells when compared to NI target cells. Most functional responses were observed in activation, homing, and cytotoxicity markers across all conventional and non-conventional T cell subsets, defining in great depth the characteristics of baseline T-CMI to S. Typhi.

**Discussion:**

This high-dimensional approach showed a complex interaction between distinct cell subsets with multifunctional properties and extended previous insights into the baseline T-CMI to *S.* Typhi. This will help guide future investigations of immunity in healthy individuals following infection or vaccination.

## 1 Introduction

*Salmonella enterica* serovar Typhi (*S.* Typhi) is a Gram-negative, human-restricted enteric bacterium that causes typhoid fever, an invasive disease with high morbidity and mortality, predominantly in low- and middle-income countries across Africa, Asia and South America (1). Complex immune responses are elicited against this intracellular pathogen, where T cell responses play an essential role for an effective host immunity. Human studies on *S.* Typhi infection and vaccination have contributed to the current understanding of host immune responses to this pathogen (2–19). In particular, controlled human infection model (CHIM) studies, in which volunteers are challenged with wild-type (wt) *S.* Typhi have been particularly valuable for the detailed characterization of *S.* Typhi-specific immune responses (20–30).

Association with protection from typhoid disease has been described in monocytes and dendritic cells, which show activation profiles after being exposed to *S.* Typhi in CHIM studies (24). These studies have also shown that classical and intermediate monocytes upregulate innate bacterial receptors (TLR4, TLR5, and mannose receptor), activation markers (HLADR, CD274, and CD86) and inflammatory molecules (TNFα) before and after exposure to wt *S.* Typhi in individuals who developed typhoid disease (28). In addition, monocytes showed a higher expression of molecules that interact with T or B cells, ensuring the linkage from innate to adaptive responses (28). Regarding the induction of B cells and antibody production in CHIM studies, B cells have shown a decrease in individuals who developed typhoid disease, while antibody avidity was reported to be directly related to protection from disease (31). Moreover, specific plasma cells expressing intestinal homing markers were induced after vaccination with the licensed oral typhoid vaccine Ty21a (25). Additionally, a decrease in highly activated circulating CD8^+^ mucosal-associated invariant T cells (MAIT) was associated with typhoid disease following *S.* Typhi challenge in participants who developed typhoid disease (19, 23). However, these studies did not reveal associations of the baseline responses before challenge with the development of disease.

In contrast, CHIM studies have shown an association of baseline levels of *S.* Typhi-responsive CD8^+^ T memory cells with protection or a higher risk of developing disease depending on the inoculum dose (21, 29). The most relevant circulating T cell subsets associated with the development of typhoid disease were T effector memory (T_EM_) and T_EM_ re-expressing CD45RA (T_EMRA_), with a minor involvement of T central memory (T_CM_) cells in protection (21).

CHIM studies have also assessed the functionality of CD4^+^ T regulatory cells (T_reg_) after exposure to *S.* Typhi (22), where a higher intestinal homing potential (expression of integrin α4β7) followed by the presence of fewer *S.* Typhi-reactive T_reg_ cells was associated to development of disease after the bacterial wt challenge. These responses might be related to the downregulation of other cell types, such as CD8^+^ T memory subsets (22). Moreover, circulating T follicular helper cells (cT_FH_) cells were increased in individuals who did not develop typhoid disease after Ty21a vaccination followed by wt *S.* Typhi challenge, with higher cytokine responses (IL21 and IL17A) associated with cT_FH_ subtypes 2 and 17, as well as higher IgA and IgG antibody titers (27). In contrast, this study revealed higher expression of activation and homing markers in cT_FH_ cells in the individuals who developed disease, suggesting a less efficient cellular response (27).

Non-conventional T cells (e.g., mucosal associated invariant T (MAIT), T gamma delta (Tγδ), and Natural-Killer T (NKT) cells) are also likely to play a role in protection from several pathogens, including bacteria, viruses, fungi, and parasites, as previously reviewed (32–34). However, their role in typhoid disease clinical outcome remains largely undefined. Thus, we also focus the present study on the baseline responsiveness of these important cell subsets and whether their presence is associated with that of *S.* Typhi-specific conventional T cells.

The study of T cell responses depends on the initial recognition of antigens by the cognate specific T cell receptor (TCR), both in CD4^+^ and CD8^+^ T cells (35). *In vivo*, interactions between antigen-presenting cells (APC) and T cells persist until cells differentiate into effector and memory cells, depending on the cell type, their dynamics and stimulation conditions. Several studies on *S.* Typhi immunity have involved *ex vivo* experiments using a short stimulation (2–3 h) with the bacteria followed by an overnight incubation before flow cytometry staining (3, 9, 13, 19, 21–23, 27, 29, 36). However, long term antigen availability might be necessary for a better effector response of some cell subsets (35, 37–40). Therefore, in this study, we performed the traditional ~18h incubation time and added a ~40 h period to extend previous studies with *S.* Typhi and enable a more comprehensive evaluation of T-CMI responsiveness over time in various cell subsets. Herein, we studied conventional CD4^+^ and CD8^+^ T memory cells, including T_EM_, T_EMRA_, and T_CM_, CD4^+^ and CD8^+^ T stem cell memory (T_SCM_), CD4^+^ cT_FH_, CD4^+^ T_reg_, and CD8^+^ T regulatory precursors (T_rp_), as well as non-conventional CD8^+^ MAIT, Tγδ, and NKT cells. The aim of this study was to use a high-dimensional data approach to further characterize the baseline responsiveness to *S.* Typhi across multiple T cell subsets in two stimulation periods (~18 and ~40 hours), in 18 healthy unexposed volunteers. The unexpected complexity of the *S.* Typhi-specific baseline responsiveness observed in our studies across conventional and non-conventional T cell subsets sheds new light into novel immune responses to bacterial pathogens and the subsets and T cell clusters that might play a role in protection from disease development.

## 2 Materials and methods

### 2.1 Clinical samples

Eighteen healthy volunteers from the Baltimore-Washington area with no history of typhoid fever vaccination or infection were randomly included in this study. 66.6% were female, the median age was 51 years (IQR, 47.75-57.25), and 61.1% were White, 33.3% African American, and 5.6% Asian, none of them were Hispanic or Latino. Peripheral blood mononuclear cells (PBMC) were isolated from whole blood and cryopreserved in liquid nitrogen until used in these studies. This study involving human participants was reviewed and approved by the University of Maryland Baltimore Institutional Review Board. All participants provided written informed consent to participate in this study.

### 2.2 Generation and infection of autologous target cells

Autologous Epstein-Barr virus (EBV)-transformed B lymphoblastoid cell lines (B-LCL) were generated from each volunteer’s PBMC, using EBV supernatant from the B95–8 cell line (ATCC CRL1612) and cyclosporine (0.5 mg/mL, Sigma-Aldrich, Saint-Louis, MO, US) as previously described (13, 17). Cultures were maintained for 2–4 weeks before infecting the cells with wild-type *S.* Typhi strain ISP1820 at a multiplicity of infection (MOI) of 10:1. Cells were incubated for 3 h at 37 °C in antibiotic-free RPMI 1640 (Gibco, ThermoFisher Scientific, Waltham, MA, US). After incubation, cells were washed three times with RPMI containing a high concentration (150 µg/mL) of gentamycin (Gibco) and cultured overnight in cRPMI (RPMI supplemented with 10% FBS [Geminibio, West Sacramento, CA, US], L-glutamine 20 mM [Gibco], HEPES 0.1 M [Gibco], penicillin/streptomycin [Sigma-Aldrich] 100,000 IU and 100 mg per L, and gentamycin [Gibco] 50 µg/mL). *S.* Typhi infection was confirmed by flow cytometry after staining with anti-CSA-1 (*Salmonella* common structural antigen 1; Kirkegaard & Perry Laboratories, Gaithersburg, MD, US) as previously described (9). Finally, B-LCL were gamma irradiated (6,000 rad) before co-culture with PBMC, where they served as antigen-presenting cells (APC). Human B cells express MHC class II molecules to present antigenic peptides to CD4^+^ T cells (41) and MHC class I molecules to present antigenic peptides to cytotoxic CD8^+^ T cells (42). Of note, *Salmonella* bacteria were found to infect human B cells (43) and we demonstrated by electron microscopy in our system that *S*. Typhi was present in the vacuoles of autologous EBV-transformed cells (17). An advantage of this system is that it is likely to allow the presentation of multiple diverse *S.* Typhi antigens/epitopes.

### 2.3 T cell stimulation by autologous APC (cell co-culture)

As previously described (21, 27, 29, 36), PBMC were thawed and resuspended to 0.5X10^6^/mL in cRPMI, and rested them overnight at 37 °C and 5% CO_2_ before stimulation with *S.* Typhi (ST)-infected target cells, at an effector:stimulator ratio of 7:1 (44) (total culture volume of 0.5 mL/tube containing 5X10^6^/mL PBMC), in the presence of anti-CD107a (metal conjugated; Standard Biotools, Markham, Ontario, CA) monoclonal antibody (mAb); non-infected (NI) target cells were used as negative controls. *Staphylococcus* enterotoxin B (SEB, 10 µg/mL) was used as a positive control in every experiment.

For the 18 h set of data, after three hours of incubation at 37 °C and 5% CO_2_, GolgiStop™ (0.5 µl/mL; Monensin; BD, Franklin Lakes, NJ, US) and GolgiPlug™ (0.5 µl/mL; Brefeldin A; BD) were added to the cell cultures and incubations continued overnight to allow cytokines/chemokines to accumulate intracellularly before permeabilization and staining for mass cytometry measurements (45, 46). For the 40 h cultures, PBMC were stimulated with *S.* Typhi-infected target cells and cultures continued for ~27 h before GolgiStop™ (BD) and GolgiPlug™ (BD) were added. Cultures were followed by an overnight incubation before staining.

### 2.4 Cytokine determination in cell culture supernatants

Since we hypothesized that the cytokine milieu after a prolonged incubation period (40 h) might affect the responses observed in significant clusters in the various cell subsets, we measured the concentration of cytokines in cell culture supernatants. At ~27 h of incubation, before adding GolgiStop™ (BD) and GolgiPlug™ (BD) to the 40 h cell cultures, tubes were centrifuged at 500 x g for 5 min; 100 µL of supernatant was collected from each tube and frozen at -80 °C until further use. A customized LEGENDplex™ (BioLegend, San Diego, CA, US) human inflammation panel was used to determine 13 cytokines/chemokines (IL1β, IL2, IL6, IL8, IL10, IL12p70, IL17A, IL18, IL23, IL33, IFNγ, TNFα, and CCL2), following the manufacturer’s instructions. Data were acquired as FCS 3.0 files using an LSR II Flow Cytometer (BD) and analyzed using the LEGENDplex™ Data Analysis Software Suite (BioLegend) to obtain each cytokine’s concentration. Net differences were calculated by subtracting the concentrations found in non-infected target cell culture supernatants from those measured in ST-infected cultures, using GraphPad Prism software (version 11.0.2, GraphPad Software LLC, Boston, MA, US).

### 2.5 Mass cytometry

PBMC were stained for mass cytometry analysis as previously described (3, 19, 27, 47). Briefly, cells were barcoded using a 4-choose-2 barcode strategy with anti-CD45 mAb tagged with 89Y, 195Pt, 196Pt and/or 198Pt (see table in **Supplementary Figure 2**), followed by a live/dead cell staining with cisplatin (194Pt) and a 20 min incubation with human Fc receptor blocker IgG. Cells were stained for surface markers and later fixed and permeabilized for intracellular staining, using the 52-monoclonal antibody panel (**Supplementary Table 1**). Cells were stained with an Ir191/193 DNA intercalator for cell detection and re-suspended in EQ4 normalization beads (Standard Biotools) before data acquisition using a Helios™ mass cytometer (Standard Biotools). Then, the CyTOF Software (version 7.1, Standard Biotools) was used to normalize signals and convert data into the Flow cytometry standard (FCS) 3.0 format. FCS files were imported into FlowJo Software (version 10.10.0, BD) for quality control through the Peak Extraction and Cleaning Oriented Quality Control (PeacoQC) plugin for FlowJo (version 1.5) (48), where the input mean absolute deviation (MAD) was established at 8.00 and the input isolation tree (IT) parameter was set at 0.80. An example of this analysis is shown in **Supplementary Figure 1**. The “PeacoQC Good Events” were exported into a new workspace to remove doublets, debris and calibration beads, following the gating strategy shown in **Supplementary Figure 2**. After gating on live cells, samples were debarcoded into the different stimuli (non-infected, *S.* Typhi-infected, media negative control, or SEB positive control) and time points (18 or 40 h) for further analysis.

### 2.6 Supervised analyses

Total CD3^+^ T cells were gated out of the total lymphocytes. Conventional CD4^+^ and CD8^+^ T cell subsets were gated out of the CD3^+^ subset, including T central memory (T_CM_), T effector memory (T_EM_), T effector memory re-expressing CD45RA (T_EMRA_), T stem memory cells (T_SCM_), CD4^+^ T regulatory cells (T_reg_), circulating CD4^+^ T follicular helper cells (cT_FH_), and CD8^+^ T regulatory precursors (T_rp_), as well as non-conventional T cell subsets: CD8^+^ mucosal associated invariant T cells (MAIT), gamma delta T cells (T_γδ_), and Natural-Killer T cells (NKT), according to the gating strategies shown in **Supplementary Figure 3**. Cytokines/chemokines, activation, homing, and cytotoxicity markers were manually gated out of each cell subset. Comparisons between ST-infected and NI target cells were evaluated with Wilcoxon matched-pairs signed rank tests, using GraphPad Prism software.

### 2.7 Unsupervised analyses

Equivalent nodes of each cell subset (e.g., CD8^+^ T_CM_) were selected for concatenation in separate FCS files for the 18 h and 40 h analyses. Event numbers were down sampled for multidimensional reduction analyses to ensure equal contribution of each sample after file concatenation. Then, Uniform Manifold Approximation and Projection FlowJo plugin (UMAP, version 4.1.1) was used to perform dimensionality reduction using a Nearest Neighbors (NN) parameter of 45 and Minimal distance of 0.1. Unsupervised clustering was performed using the PhenoGraph plugin for FlowJo (version 4.05), with an optimal K value (K = 40-100) for clustering analysis of high-dimensional data. The Euclid plugin for FlowJo (version 0.8) was used to verify the quality of the clustering, obtaining an optimal Taylor index (50.14-139.08) for each cell subset. PhenoGraph clusters were then visualized on the initial UMAP to create a reference map of all automatically detected cell subsets. Afterwards, the ClusterExplorer tool within FlowJo was used to generate heatmaps and perform hierarchical clustering in each cell subset. From each volunteer’s ClusterExplorer bar graphs, we exported the number of events for each cluster, both from NI and ST-infected target cells. Cluster events per volunteer were analyzed with Wilcoxon matched-pairs signed rank tests, using GraphPad Prism. Clusters with significant differences (p<0.05) between NI and ST-infected target cells were selected for supervised validation, which was performed on the original FCS files containing all events. The supervised gating utilized the most intensely expressed markers according to the corresponding ClusterExplorer heatmap. Outliers were removed from NI and ST-infected target cells data using the ROUT method from GraphPad Prism (49). “Clean data” were compared with Wilcoxon match-pairs signed rank tests, using GraphPad Prism, and significant differences were defined as p<0.01. The percentage change in each cluster was calculated using a conventional formula (( ST−NI/NI)*100), and median with IQR were calculated; outliers were excluded from further analyses. The unsupervised analysis workflow can be visualized in **Supplementary Figure 4**.

### 2.8 Statistical analyses

#### 2.8.1 Paired comparisons

In all supervised and unsupervised analyses, comparisons between ST-infected and NI target cells were evaluated by Wilcoxon matched-pairs signed rank tests after outlier removal using the ROUT (robust regression and outlier removal) method (49). Analyses were performed using GraphPad Prism software. Significant differences were defined as p<0.01.

#### 2.8.2 Correlation analyses

Clusters with significant differences (p<0.01) between NI and ST-infected targets after 18 h (n=39) or 40 h (n=55) incubation were selected to perform non-parametric Spearman correlation analyses. Labels indicate the cell subsets (e.g., CD4TSCM) followed by the cluster number. The arithmetic difference between cytokine concentrations found in NI and ST-infected target cell culture supernatants (n=10) were also included in the Spearman’s analyses. Significant differences between clusters and/or cytokines were defined as p<0.01.

## 3 Results

### 3.1 Supervised analyses

Initially, we performed supervised analyses to assess the reproducibility and consistency of results from our previous studies. **Table 1** shows the proportion of every cell subset studied in the CD3^+^ compartment, both at 18 h (panel A) and 40 h (panel B) incubation. Interestingly, there is a notable increase in the proportion of CD4^+^ and CD8^+^ T_SCM_ at 40 h, whereas the other cell subsets remain largely unchanged between these two culture time points. We gated on several activation (CD28, CD39, CD69, CD134, CD137, CD154, HLADR, ICOS, PD1, Tim3) proliferation (CD71) homing (CCR6, CCR7, CCR9, CD62L, integrin α4β7), and cytotoxicity (CD107a, GranzymeB, KIR) markers, as well as cytokines/chemokines (IL2, IL10, IL17A, IL21, IFNγ, TNFα) to assess cell functionality. We evaluated these markers in activated cells (those expressing CD69) and found significant increases in the percentage of cells responding to ST-infected target cells when compared to NI, across all cell subsets and in both incubation periods (18 and 40 h). **Figure 1** shows the increase in the percentage of IFNγ- and CCR9-expressing cells in selected T memory, T regulatory, T helper, and T cytotoxic subsets, as examples of the data obtained with these supervised gating strategies on the previously defined conventional and non-conventional T cell subsets. Data for additional cytokines and homing markers are shown in **Supplementary Figures 5–7**.

**Table 1:** Summary of significant changes observed in cell co-cultures after exposure to autologous *S.* Typhi-infected target cells.

Panel A: 18 h cultures							
Cell subset	Percentage from CD3+ T cells (median, [IQR])	Antigen / MHC - T receptor interaction	Total significant % change per subset	Main clusters	Percentage of representation in concatenated file	Percentage change (median, [IQR])	Cluster phenotype
NKT	1.2 [0.7-1.7]*	Lipids, glycolipids and Glycosphingolipids / CD1d** - Vα24-Jα18-Vβ11 TCR & TCR-independent, cytokine-driven activation	45.5%	↑ Cluster 4	11.5%	77.3 [33.5-151.8]	CD161+ CD45RO+ **CD95+ CD107a+ CD69+ GZMB+ HLADR+** CD27+
				↑ Cluster 2	14.5%	35.9 [20.9-143.5]	KIR+ **GZMB+** CD45RO+ **CD95+ CCR9+ CXCR3+ HLADR+ CD62L+ CCR7+ CD107a+** CD27+ IL21+
				↑ Cluster 15	5%	35.2 [20.5-47.9]	KIR+ **CD95+** CD45RA+ **CCR7+**
				↑ Cluster 6	7.5%	26.3 [16.1-52.7]	CD161+ **CD95+ CD107a+** CD45RA+
				↑ Cluster 5	7%	22.5 [15.1-46.2]	**GZMB+** CD57+ KIR+ CD45RA+
MAIT	1.4 [0.9-2.6]	Riboflavin metabolites / MR1 - semi-invariant TCR (Vα7.2-Jα33/12/20 chain)	40.5%	↑ Cluster 3	13.5%	587.5 [311-1302]	**CD69+ CD107a+** CD45RO+ **GZMB+**
				↑ Cluster 11	8%	203.9 [124.2-347.3]	CD45RO+ **CD107a+ CD95+** KIR+ **CD69+ GZMB+**
				↑ Cluster 10	4%	28.6 [22.9-74.9]	CD45RO+ **CD107a+ CD95+ HLADR+** KIR+
				↑ Cluster 9	15%	16.1 [7.6-32.7]	CD45RO+ **CD107a+ CD95+**
CD4+ T_SCM_	4.2 [2.2-8.7]	Cognate Protein/Peptide / MHC II - TCR	35.0%	↑ Cluster 2	8%	33.3 [17.7-108.5]	**HLADR+** KIR+ **CD107a+** TNFα+
				↑ Cluster 6	14%	32 [15.2-64.5]	**HLADR+** KIR+ CD127+
				↑ Cluster 4	6%	25.4 [7.7-52.9]	KIR+ **CD107a+**
				↑ Cluster 9	7%	20.4 [10.6-41.2]	KIR+ **HLADR+**
CD8+ T_EMRA_	5.5 [4.1-8.6]	Cognate Protein/Peptide / MHC I - TCR	33.0%	↑ Cluster 2	7.5%	31 [15.4-97.4]	CD161+ **GZMB+ CD107a+ CD95+ HLADR+** CD27+
				↑ Cluster 11	12.5%	27.8 [8.6-49.6]	**CCR7+** KIR+ **GZMB+ CXCR3+ CCR9+ CD107a+** CD27+
				↑ Cluster 14	8.5%	17.1 [5.5-27.2]	CD27+ **CCR7+ HLADR+** KIR+
				↑ Cluster 10	4.5%	14.6 [5-40.2]	KIR+ **CCR7+ CXCR3+ GZMB+ HLADR+**
CD4+ T_EM_	33.4 [16.6-35.2]	Cognate Protein/Peptide / MHC II - TCR	32.0%	↑ Cluster 10	6%	38.1 [20-69.9]	**CD95+** CD27+ **HLADR+ CCR7+ α4β7+** KIR+
				↑ Cluster 15	2.5%	36 [14.4-49.6]	**CD95+** CD27+ KIR+ **HLADR+ CCR7+**
				↑ Cluster 6	6%	27 [12.6-32.9]	**CD95+** CD27+ **HLADR+** KIR+
				↑ Cluster 13	6%	25.7 [7.9-33.4]	**CD95+** CD27+ **HLADR+** CD28+ KIR+
				↑ Cluster 14	5.5%	24.4 [7.3-41.5]	**CD95+** CD27+ KIR+ CD28+ **CCR7+**
				↑ Cluster 16	2%	22.3 [18.1-35.1]	**CD95+** CD27+ KIR+ CD28+ CD161+ **CCR7+**
				↑ Cluster 12	4%	20.9 [8.3-31.4]	**CD95+** CD27+ **HLADR+** KIR+ CD28+
CD4+ T_reg_	1.9 [1.5-3.9]	Cognate Protein/Peptide / MHC II - TCR	25.0%	↑ Cluster 1	12%	10 [3.4-18.1]	**CD95+** CD45RO+ **HLADR+** CD27+ CD39+ KIR+ **ICOS+ CCR7+**
				↓ Cluster 6	10%	-10.4 [(-17.6) – (-2.9)]	**CD62L+** CD27+ **CD95+** CD45RO+ **ICOS+ HLADR+**
				↓ Cluster 13	3%	-7 [(-14.8) – (-2.9)]	**CD62L+** CD27+ **CD95+** CD45RO+
CD8+ T_EM_	6.2 [4.7-8.4]	Cognate Protein/Peptide / MHC I - TCR	23.5%	↑ Cluster 9	6.5%	77.6 [44.3-169.8]	**HLADR+** KIR+ **GZMB+ CD95+ CCR9+ CD107a+ CXCR3+ CCR7+**
				↑ Cluster 10	10%	74.3 [16.4-171.4]	KIR+ **CXCR3+ GZMB+ CCR9+**
				↑ Cluster 8	7%	66.8 [28.4-80.6]	**CD95+ HLADR+** KIR+ **GZMB+** CD27+
CD4+ T_EMRA_	6 [3.8-8.4]	Cognate Protein/Peptide / MHC II - TCR	19.0%	↑ Cluster 11	19%	15.1 [10.7-19.6]	**CCR7+** KIR+ **CXCR3+ CCR9+**
CD8+ T_rp_	1 [0.9-1.3]	Cognate Protein/Peptide / MHC I - TCR	15.5%	↑ Cluster 12	6%	43.1 [35.8-74.5]	**CD95+** CD45RO+ **CD62L+** KIR+ **GZMB+ CD107a+**
				↑ Cluster 3	9.5%	41 [22.1-69.4]	**CD107a+** KIR+ **CD95+** TNFα+
CD4+ cT_FH_	2 [1.5-3.3]	Cognate Protein/Peptide / MHC II - TCR	13.5%	↓ Cluster 8	3.5%	-13.6 [(-23.1) – (-6.6)]	**CD62L+** CD27+ **CD95+** **CCR7+** CD161+ CD28+ KIR+
				↓ Cluster 3	10%	-10.4 [(-15.3) – (-6.5)]	CD27+ **CD62L+ CCR7+** **CD95+ HLADR+** CD127+ **CXCR3+ ICOS+** CD28+ KIR+
Tγδ	2 [1-3.1]	Phosphoantigens / MHC-unrestricted - Vγ9Vδ2 TCR	10.5%	↑ Cluster 6	3%	53 [35.3-93.3]	**HLADR+ CD107a+** KIR+ **CCR9+ GZMB+ CXCR3+ CD95+** TNFα+
				↑ Cluster 5	7.5%	39.2 [23.8-82.3]	CD45RO+ CD161+ **CD95+ HLADR+ CD69+ CD107a+** CD27+ CD127+ **CXCR3+ GZMB+ CD62L+**
CD4+ T_CM_	8.9 [4.8-11.8]	Cognate Protein/Peptide / MHC II - TCR	8.0%	↑ Cluster 2	3.5%	47.5 [9.5-131]	**HLADR+ CD107a+** TNFα+ **CD95+** KIR+ **CCR9+**
				↑ Cluster 11	4.5%	14.5 [4.2-23]	**CD95+** CD27+ KIR+ CD28+ **HLADR+**
CD8+ T_CM_	1.1 [0.5-1.7]	Cognate Protein/Peptide / MHC I - TCR	–	–	–	–	–
CD8+ T_SCM_	1.4 [0.7-2.4]	Cognate Protein/Peptide / MHC I - TCR	–	–	–	–	–

Panel B: 40 h cultures							
Cell subset	Percentage from CD3+ T cells (median, [IQR])	Antigen / MHC - T receptor interaction	Total significant % change per subset	Main clusters	Percentage of representation in concatenated file	Percentage change (median, [IQR])	Cluster phenotype
MAIT (cont)	1.3 [0.6-2.1]	Riboflavin metabolites / MR1 - semi-invariant TCR (Vα7.2-Jα33/12/20 chain)	73.0%	↑ Cluster 8	5.5%	10,186 [6,134-30,059]	**CD25+ GZMB+ CD69+ ICOS+ CD95+ HLADR+ CD107a+** IFNγ+
				↑ Cluster 7	11.5%	7,644 [4,678-19,429]	**CD25+ GZMB+ CD69+ ICOS+** IFNγ+ **CD95+**
				↑ Cluster 9	13.5%	4,101 [1,149-9,352]	**CD25+ GZMB+ CD69+ ICOS+ CD95+ HLADR+ CD107a+** CD45RO+
				↑ Cluster 10	2%	3,627 [1,388-45,150]	**CD25+ GZMB+ CD69+ ICOS+ CD95+ HLADR+ CD107a+** CD57+
				↑ Cluster 11	2.5%	1,921 [824.1-3,972]	**CD69+ GZMB+ CD107a+** IFNγ+
				↑ Cluster 1	17%	566.7 [316.2-1001]	**CD69+** CD45RO+ **CD95+ HLADR+ GZMB+ CD107a+**
				↑ Cluster 6	11.5%	79.2 [28-219.3]	CD45RO+ **CD95+ HLADR+** KIR+
				↓ Cluster 12	4%	-61.7 [(-77.6) – (-50.2)]	CD45RO+ **CD95+ CD69+** CD127+
				↓ Cluster 4	5.5%	-49.9 [(-66.6) – (-28.2)]	CD45RO+ **CD95+ HLADR+** CD127+ KIR+
Tγδ	1.8 [0.8-3]	Phosphoantigens / MHC-unrestricted - Vγ9Vδ2 TCR	56.5%	↑ Cluster 8	8%	582.1 [386.3-1909]	**GZMB+ CD25+ CD95+ HLADR+ CD69+** CD45RO+
				↑ Cluster 10	2%	184.3 [94.1-253.6]	CD57+ **CD25+**
				↑ Cluster 9	5%	78.3 [28.6-158.9]	CD45RA+ **CD62L+ CD95+ HLADR+** CD161+ **GZMB+ CD107a+**
				↑ Cluster 3	13%	78.1 [40.6-137.6]	CD45RO+ CD161+ **GZMB+ CD95+ CD69+ HLADR+ CD62L+ CD107a+**
				↑ Cluster 4	12%	74.7 [24.9-147.4]	**GZMB+** CD57+ CD45RA+ KIR+ **CD107a+ CXCR3+**
				↑ Cluster 11	9.5%	43.6 [28.2-82.8]	**GZMB+** KIR+ CD45RA+ **CD107a+ CXCR3+ HLADR+ CD95+ CCR9+**
				↑ Cluster 5	7%	41.6 [18.8-94]	**CD62L+** CD45RA+ CD27+ **CCR7+ CD95+ HLADR+** CD127+ **CXCR3+ GZMB+**
CD8+ T_EM_ (cont)	6.3 [4.1-7.6]	Cognate Protein/Peptide / MHC I - TCR	35.5%	↑ Cluster 13	5%	425.6 [174.3-2147]	**CD25+ GZMB+ CD95+ ICOS+**
				↑ Cluster 7	5%	222.5 [142.8-447.7]	**CD25+ CD95+ HLADR+ CD107a+**
				↑ Cluster 3	6.5%	89.1 [14.5-209.3]	**CD95+ HLADR+** CD27+ CD127+ **CXCR3+ GZMB+**
				↑ Cluster 14	3%	73.5 [12.7-160.4]	KIR+ **GZMB+ CD95+ HLADR+ CXCR3+**
				↑ Cluster 15	5.5%	71.1 [7.1-133.2]	**CD95+** KIR+ **CXCR3+ CCR7+**
				↑ Cluster 11	3.5%	41.3 [14.4-66]	CD57+ **GZMB+** KIR+ **CD95+**
				↓ Cluster 8	7%	-17.3 [(-27.8) – (-9.3)]	**CD95+ HLADR+** CD27+ CD127+
NKT	1.1 [0.7-1.6]*	Lipids, glycolipids and Glycosphingolipids / CD1d** - Vα24-Jα18-Vβ11 TCR & TCR-independent, cytokine-driven activation	35.0%	↑ Cluster 5	5.5%	259.9 [168.8-558.6]	**GZMB+ CD25+ CD69+ CD95+** CD45RO+ **CD107a+**
				↑ Cluster 8	4.5%	154.7 [62.1-268.7]	**HLADR+** KIR+ **CD95+ CD107a+** TNFα+ **CCR7+ CD25+ CCR9+** CD71+ CTLA4+
				↑ Cluster 2	14%	84.6 [32.1-134.9]	CD45RO+ CD161+ **CD95+ HLADR+ CD69+ GZMB+**
				↑ Cluster 12	8.5%	37.9 [16.3-73.2]	**HLADR+ CCR7+** KIR+ **CD107a+ CCR9+ CXCR3+ GZMB+ CD95+** CD45RA+ CD154+ **CD62L+** IL21+
				↑ Cluster 13	2.5%	31.6 [18.3-57.1]	**HLADR+** KIR+ **CD107α+** TNFa+ **CD95+** CTLA4+ CD45RO+ CD71+ **CCR9+ α4β7+**
CD8+ T_CM_	1.2 [0.5-2.1]	Cognate Protein/Peptide / MHC I - TCR	33.5%	↑ Cluster 6	5.5%	129.2 [87.7-726.6]	**CD25+ CD95+ HLADR+ ICOS+ CD69+ GZMB+**
				↑ Cluster 7	6%	76.7 [44.1-98.7]	**CD95+ HLADR+** CD27+ CD127+ **ICOS+ CCR7+**
				↑ Cluster 8	8.5%	67.9 [13.7-104.5]	**CD95+ HLADR+** CD27+ KIR+ CD127+ **ICOS+**
				↑ Cluster 3	11%	62.6 [33.9-116.2]	**CD95+ HLADR+** CD27+ CD127+ **CXCR3+ ICOS+ CCR7+** CD28+
				↑ Cluster 14	2.5%	43.92 [18.8-134.9]	**CD95+ HLADR+** KIR+ CD27+ **ICOS+ CXCR3+**
CD4+ T_CM_	8.2 [4.6-13.7]	Cognate Protein/Peptide / MHC II - TCR	31.5%	↑ Cluster 13	8.5%	55.4 [41.1-120.8]	**CD95+ CD25+ HLADR+** CD27+ **ICOS+** KIR+
				↑ Cluster 3	13.5%	34.7 [12.8-73.1]	**CD95+ HLADR+** CD27+ **ICOS+** CD127+ CD161+ **CCR7+** CD28+ **CD107a+** KIR+ **CXCR3+** CD58+
				↑ Cluster 2	5%	21.3 [12.8-31.8]	**CD95+ HLADR+** CD27+ **ICOS+** KIR+ **CCR7+ CD107a+** CD28+
				↑ Cluster 8	4.5%	19 [13.7-34.1]	**CD95+ HLADR+** CD27+ KIR+ CD28+ **CD107a+**
CD8+ T_SCM_	3.3 [1.3-5.8]	Cognate Protein/Peptide / MHC I - TCR	27.0%	↑ Cluster 4	8%	46.8 [21-82.5]	**HLADR+ ICOS+ CXCR3+** CD127+
				↑ Cluster 5	3%	46.3 [26.5-70.2]	**HLADR+ CD25+ GZMB+** KIR+
				↑ Cluster 8	7.5%	29.9 [13.3-68.2]	**HLADR+** KIR+ **CD107a+ CXCR3+**
				↑ Cluster 12	5.5%	16.7 [6.1-26.8]	**HLADR+ CD107a+** KIR+
				↓ Cluster 10	3%	-36 [(-44.6) – (-21.7)]	**HLADR+ α4β7+**
CD4+ T_reg_	2.3 [1.4-3.4]	Cognate Protein/Peptide / MHC II - TCR	20.0%	↑ Cluster 8	10%	88.2 [40.1-148.6]	**CD95+** CD45RO+ **HLADR+ CD62L+** CD27+ **ICOS+** CD161+ KIR+
				↓ Cluster 1	10%	-31.6 [(-50.2) – (-18.1)]	**CD95+** CD45RO+ **HLADR+ CD62L+** CD27+ **ICOS+** CD39+ CD134+
CD4+ T_EMRA_	6.8 [3.9-10.8]	Cognate Protein/Peptide / MHC II - TCR	16.5%	↑ Cluster 3	8.5%	98.9 [57.6-212.7]	**CD95+ CD25+ HLADR+** CD27+ **CCR7+ ICOS+**
				↑ Cluster 7	8%	38.6 [13.4-52.4]	**CCR7+** CD27+ **HLADR+ CD95+** TNFα+ **CD69+**
CD8+ T_EMRA_	5.2 [3.4-9.2]	Cognate Protein/Peptide / MHC I - TCR	14.0%	↑ Cluster 7	3%	115 [37.7-142.2]	**CD25+ GZMB+ CD95+ HLADR+**
				↑ Cluster 12	6%	36.9 [10.6-46.9]	**CCR7+** CD27+ **HLADR+ CD95+**
				↑ Cluster 16	5%	12.5 [8.7-42.3]	KIR+ **CCR7+ HLADR+ CD95+**
CD4+ cT_FH_	2 [1.2-3.1]	Cognate Protein/Peptide / MHC II - TCR	11.5%	↑ Cluster 10	9%	65 [20.5-99.1]	**CD62L+ CD95+** CD27+ **CCR7+ HLADR+ CD25+ ICOS+** KIR+ **CD107a+** CD28+
				↑ Cluster 7	2.5%	51.3 [19.6-82.9]	**CD62L+ CD25+ CD95+ ICOS+ HLADR+** CD27+
CD4+ T_EM_	21.6 [14.7-33]	Cognate Protein/Peptide / MHC II - TCR	9.5%	↑ Cluster 5	5%	154.9 [89.9-233.2]	**CD95+ HLADR+ CD25+ ICOS+ CD69+ GZMB+**
				↑ Cluster 7	4.5%	55.8 [19.4-104.5]	**CD95+ HLADR+ CD25+** CD27+ KIR+ **ICOS+**
CD4+ T_SCM_	5.3 [2.5-11.1]	Cognate Protein/Peptide / MHC II - TCR	8.5%	↑ Cluster 3	8.5%	91.1 [41.4-187.4]	**CD25+ HLADR+** KIR+ **ICOS+**
CD8+ T_rp_	0.8 [0.6-1.4]	Cognate Protein/Peptide / MHC I - TCR	8.5%	↑ Cluster 4	8.5%	142.1 [52.6-631.1]	**GZMB+ CD95+ ICOS+ CD25+ CD69+ CD107a+**

*The proportion of NKT cells is reported out of the total T cell subset (see gating strategy in **Supplementary Figure 3B**).

**In *Salmonella* infection, CD1d recognizes host self-glycosphingolipids, not bacterial antigens.

Colored letters highlight some of the most frequently found markers to be significant across cell subsets. GREEN=activation markers. PURPLE=cytotoxicity markers. RED=homing markers.

↑ Cluster showed significant (p<0.01) increases in cultures with *S. Typhi*-infected target cells than in cultures with non-infected target cells.

↓ Cluster showed significant (p<0.01) decreases in cultures with *S. Typhi*-infected target cells than in cultures with non-infected target cells.

**Figure 1:**
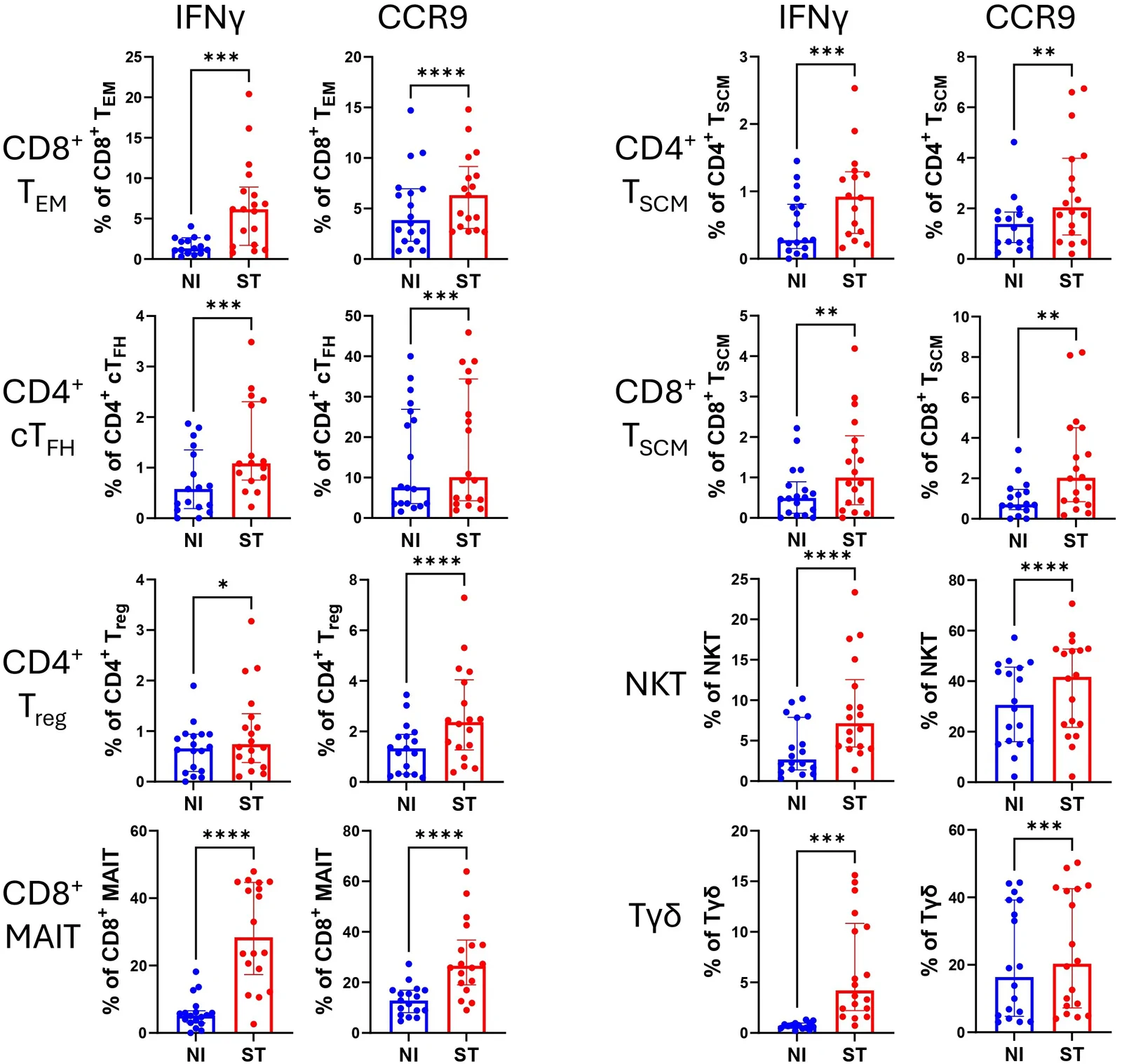
Examples of *Salmonella*-specific cytokine production (IFNγ) and gut homing marker expression (CCR9) by selected T cell subsets after ex-vivo exposure to *S.* Typhi-infected autologous targets in 18 healthy volunteers. Supervised analysis of baseline PBMC responses after an 18 h co-culture with autologous EBV-transformed lymphoblastoid B cell lines (B-LCL) showing significant differences between non-infected (NI, blue) and *S.* Typhi-infected (ST, red) targets. Medians with interquartile ranges are shown in each bar, each dot represents individual data from each participant. Comparisons between groups were evaluated with Wilcoxon match-pairs signed rank tests, using GraphPad software version 11.0.0. T_EM_, T effector memory cells. cT_FH_, circulating T follicular helper cells. T_reg_, T regulatory cells. MAIT, mucosal-associated invariant T cells. T_SCM_, T stem memory cells. NKT, Natural Killer T cells. Tγδ, gamma-delta T cells. *p<0.05, **p<0.01, ***p<0.001, ****p<0.0001.

### 3.2 Unsupervised analyses

To understand the immune system as a multifunctional entity, where activated cells might not express a unique marker but provide heterogeneous responses to a pathogen, we used a high-dimensional approach to further describe the complex baseline responsiveness to *S.* Typhi (**Supplementary Table 1**). As our CyTOF panel surpasses 50 markers to be evaluated per cell, the resulting phenotypic complexity exceeds the reach of supervised gating, making unsupervised, hypothesis-free analyses indispensable for comprehensively and reproducibly resolve the cellular landscape. Data analyses across all studied cell subsets from all volunteers showed a range of 8-61% of validated clusters with significant increases (p<0.01) in cultures with ST-infected targets compared to NI. Most differences were observed in activation (e.g., CD69, HLADR, CD95, ICOS), homing (e.g., CD62L, CCR9, integrin α4β7), and cytotoxicity (e.g., Granzyme B, CD107a) markers across conventional and non-conventional T cell subsets, as thoroughly described in the sections below. Moreover, we compiled the significant (p<0.01) data in **Table 1** and ordered all significant results according to the contribution of each cell subset to the baseline responsiveness to *S.* Typhi, where panel A shows the results concerning 18 h incubation analyses and panel B the 40 h incubation analyses.

#### 3.2.1 Conventional T cell subsets

CD8^+^ T memory cells are comprised of persistent lymphocyte subsets characterized by clonal expansion, restricted TCR repertoires, high cytotoxicity, and a wide systemic distribution, including circulating and tissue resident cells (50–52). CD4^+^ T memory cells, also widely distributed in blood and other tissues, coordinate organized humoral and/or cellular responses using a more diverse and less expanded TCR repertoire, as well as production of polarizing cytokines for their interaction with other cells (50, 52, 53). In this study, we focused on the circulating T cell subsets, resolved by a sequential gating on CD62L selectin and CD45RO to define memory populations, and on CD45RA to distinguish central memory, effector memory, and effector memory re-expressing CD45RA cells (**Supplementary Figure 3**).

##### 3.2.1.1 CD8^+^ T central memory

CD8^+^ T_CM_ are long-term memory cells with high proliferation and cytotoxic switching capacities, especially after re-stimulation with a cognate antigen (50–52). In this study, CD8^+^ T_CM_ comprised ~1% of CD3^+^ lymphocytes. After 18 h incubation, no cluster showed significant differences between cultures with ST-infected target cells and NI (**Figures 2A–C**). However, after 40 h incubation, five out of 14 clusters showed significant increases in cultures with ST-infected targets when compared to NI, accounting for ~33% of the cluster events in the concatenated file (**Figures 2D, E**; **Supplementary Figure 8**). The heatmap revealed a high expression of CD95 and HLADR in all the validated clusters, as well as CD27 and ICOS in most of these clusters (**Figures 2F, G**; **Supplementary Figure 8**).

**Figure 2:**
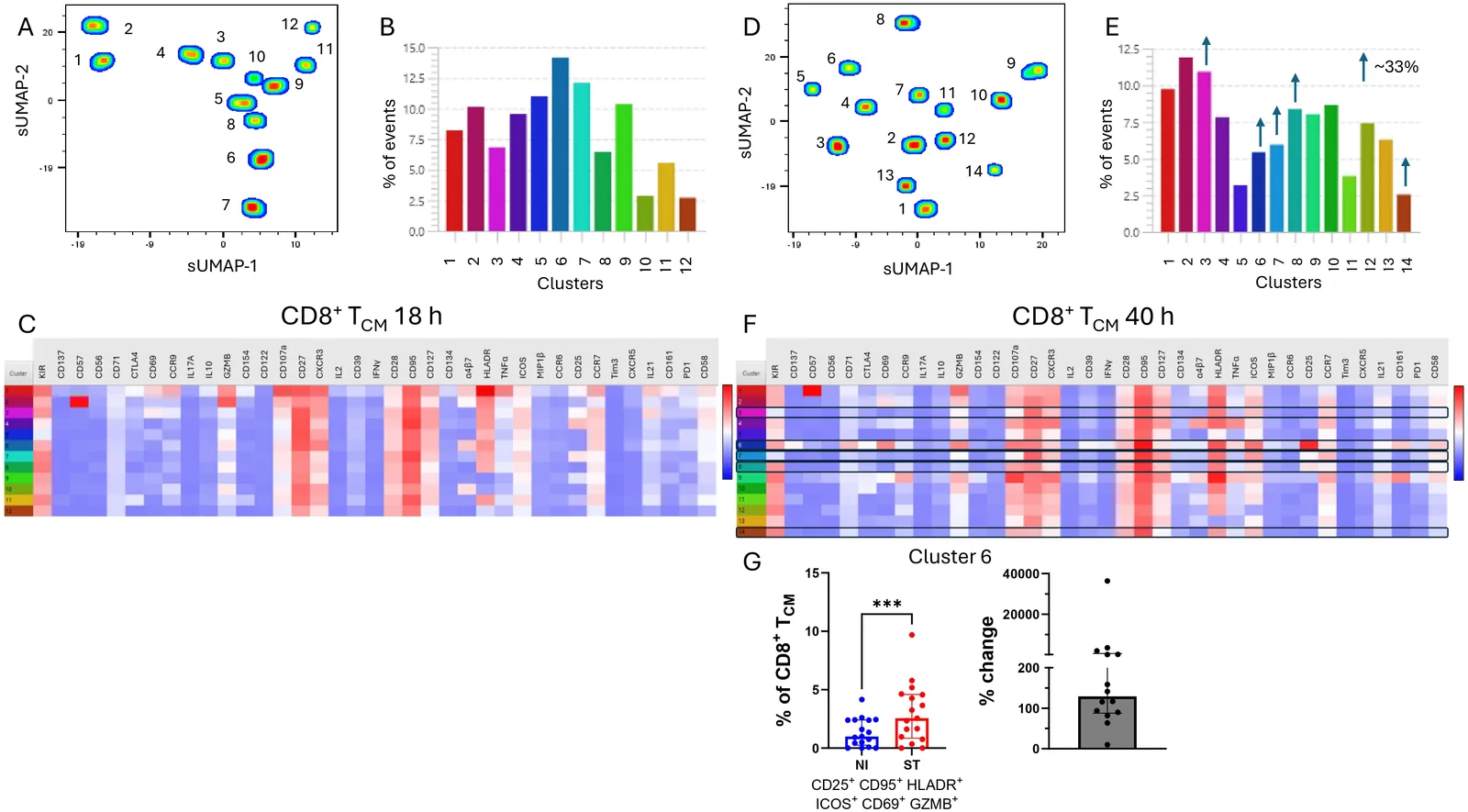
Unsupervised high-dimensional analysis of CD8^+^ T central memory (T_CM_) cells after ex-vivo exposure to *S.* Typhi-infected autologous targets in 18 healthy volunteers. PhenoGraph clusters visualized on UMAP reference maps of CD8^+^ T_CM_ cells after an 18 h **(A)** and 40 h **(D)** PBMC co-culture with autologous EBV-transformed lymphoblastoid B cell lines. Panels **(B, E)** show hierarchical clusterings of concatenated CD8^+^ T_CM_ cells after 18 h **(B)** and 40 h **(E)** incubation, respectively. Arrows show clusters with significant increases (p<0.01) after comparing non-infected (NI) and *S.* Typhi (ST)-infected targets. The percentage shown in the bar graph is the estimated representation of the changing clusters. Heatmaps were generated after clustering of concatenated CD8^+^ T_CM_ cells after 18 h **(C)** and 40 h **(F)** incubation. Clusters with significant increases are denoted by black outlined rectangles. The varying levels of intensity on the expression of each marker are shown in the blue (low) to red (high) scale. Panel **(G)** depicts the validation of a cluster showing significant differences between NI (blue) and ST (red) target cells, and the corresponding percentage change (gray), calculated using a conventional formula: $[\frac{ST-NI}{NI}]*100$. Comparisons between groups were evaluated with Wilcoxon match-pairs signed rank tests, using GraphPad software version 11.0.0. Medians with interquartile ranges are shown in each bar, each dot represents individual data from each participant [panel **(G)**]; markers selected for the supervised gating to validate the unsupervised analysis are shown below the graph. ***p<0.001.

##### 3.2.1.2 CD8^+^ T effector memory

CD8^+^ T_EM_ are preferably found in circulation and non-lymphoid tissues, responsible for first responses against cognate antigens, and characterized by high production of effector cytokines (IFNγ, TNFα) (50–52). We found that CD8^+^ T_EM_ comprised ~4.14% of CD3^+^ lymphocytes. In this analysis, three out of 12 clusters showed significant increases in cultures with ST-infected target cells after 18 h incubation, highlighting HLADR, KIR, and Granzyme B (GZMB) as markers distributed along these clusters that represented ~24% of the cluster events in the concatenated file (**Figures 3A–D**; **Supplementary Figure 9A**). Moreover, after 40 h incubation, six out of 16 clusters showed significant increases in cultures with ST-infected targets when compared to NI, also emphasizing markers such as HLADR, CD95, and GZMB in several clusters, and accounting for ~28% of the cluster events (**Figures 3E–H**; **Supplementary Figure 9B**). Interestingly, one cluster showed a significant decrease in cultures with ST-infected target cells, corresponding to ~7% of the concatenated cluster events. The markers that validated this cluster were CD95, HLADR, CD27, and CD127 (**Supplementary Figure 9B**).

**Figure 3:**
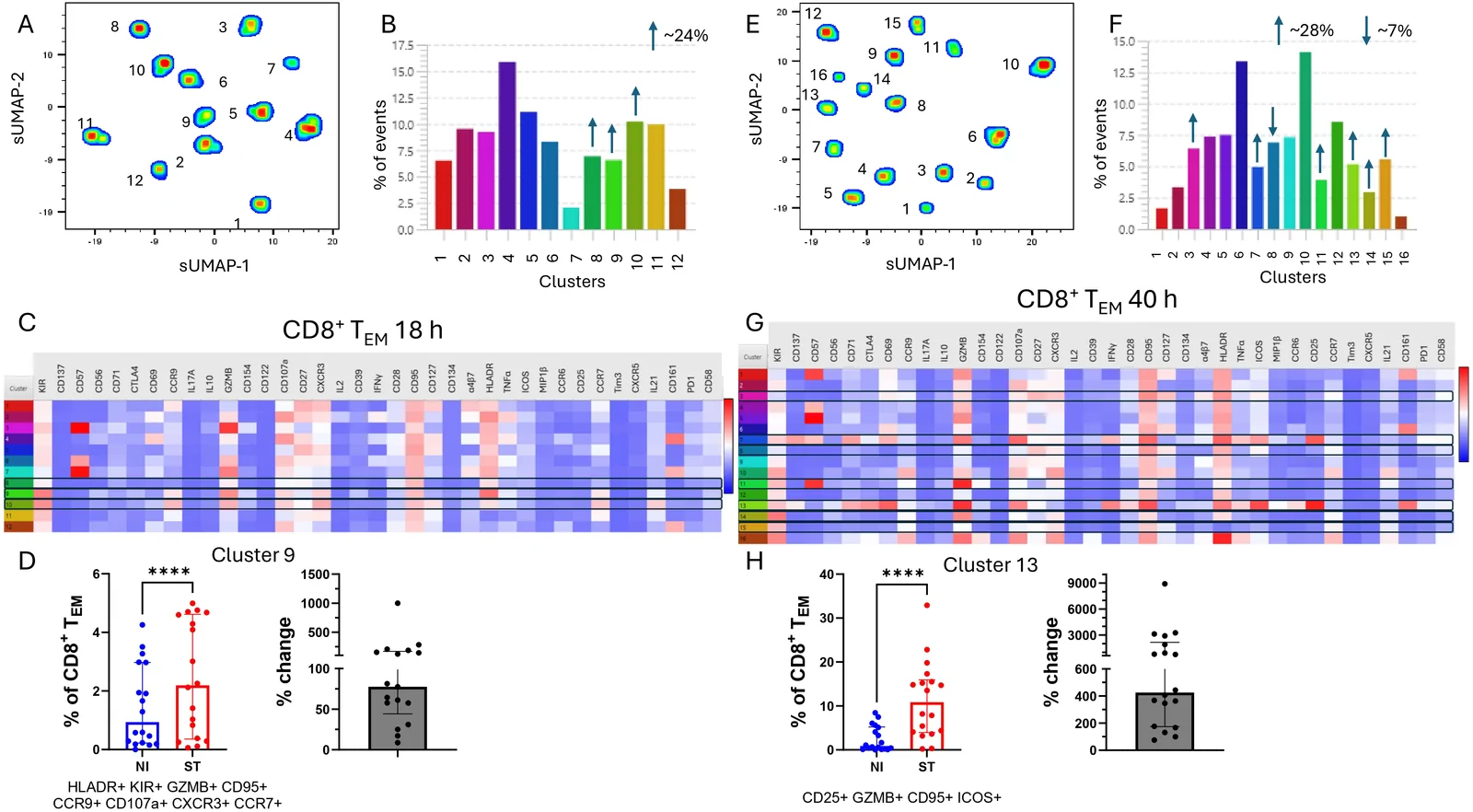
Unsupervised high-dimensional analysis of CD8^+^ T effector memory (T_EM_) cells after *ex-vivo* exposure to *S.* Typhi-infected autologous targets in 18 healthy volunteers. PhenoGraph clusters visualized on UMAP reference maps of CD8^+^ T_EM_ cells after an 18 h **(A)** and 40 h **(E)** PBMC co-culture with autologous EBV-transformed lymphoblastoid B cell lines. Panels **(B, F)** show hierarchical clusterings of concatenated CD8^+^ T_EM_ cells after 18 h **(B)** and 40 h **(F)** incubation, respectively. The arrows show clusters with significant increases or decreases (p<0.01) after comparing non-infected (NI) and *S.* Typhi (ST)-infected targets. The percentage shown within each graph is the estimated sum of all changing clusters. Heatmaps were generated after clustering of concatenated CD8^+^ T_EM_ cells after 18 h **(C)** and 40 h **(G)** incubation. Clusters with significant increases are denoted by black outlined rectangles. The varying levels of intensity on the expression of each marker are shown in the blue (low) to red (high) scale. Panels **(D, H)** depict examples of the validation of a cluster showing significant differences between NI (blue) and ST (red) target cells, and the corresponding percentage change (gray), as calculated using a conventional formula: $[\frac{ST-NI}{NI}]*100$. Comparisons between groups were evaluated with Wilcoxon match-pairs signed rank tests, using GraphPad software version 11.0.0. Medians with interquartile ranges are shown in each bar, each dot represents individual data from each participant [panels **(D, H)**]; markers selected for the supervised gating to validate the unsupervised analyses are shown below each graph. ****p<0.0001.

##### 3.2.1.3 CD8^+^ T effector memory re-expressing CD45RA

CD8^+^ T_EMRA_ are terminally differentiated and highly cytotoxic memory cells with a consistent expansion in blood and other tissues, usually related to repeated antigenic stimulation (50, 51, 54). In this study, CD8^+^ T_EMRA_ comprised ~6.2% of CD3^+^ lymphocytes. After 18 h incubation, four out of 16 clusters showed significant increases in cultures with ST-infected target cells compared to NI (**Figures 4A–D**; **Supplementary Figure 10A**), with the most expressed markers being HLADR, GZMB, and KIR in the selected clusters that represented ~33% of cluster events in the concatenated file. After 40 h incubation, three out of 16 clusters showed significant increases in cultures with ST-infected targets when compared to NI, accounting for ~14% of cluster events (**Figures 4E–H**; **Supplementary Figure 10B**). The heatmap revealed a high expression of CD95, HLADR and CCR7 in most of the validated clusters (**Figure 4G**; **Supplementary Figure 10B**).

**Figure 4:**
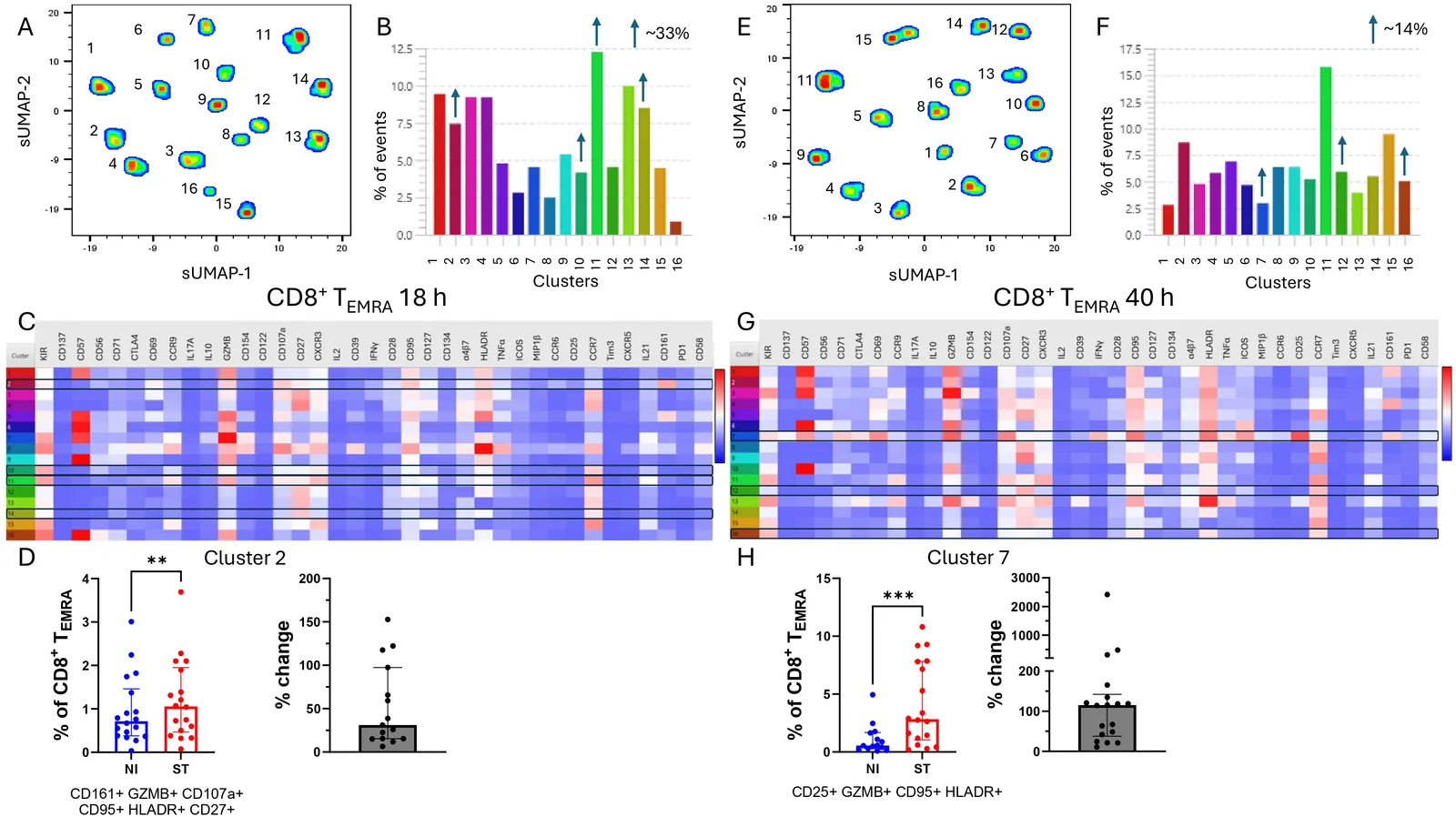
Unsupervised high-dimensional analysis of terminally differentiated CD8^+^ T effector memory cells re-expressing CD45RA (T_EMRA_) after ex-vivo exposure to *S.* Typhi-infected autologous targets in 18 healthy volunteers. PhenoGraph clusters visualized on UMAP reference maps of CD8^+^ T_EMRA_ cells after an 18 h **(A)** and 40 h **(E)** PBMC co-culture with autologous EBV-transformed lymphoblastoid B cell lines. Panels **(B)** and **(F)** show hierarchical clusterings of concatenated CD8^+^ T_EMRA_ cells after 18 h **(B)** and 40 h **(F)** incubation, respectively. The arrows show clusters with significant increases (p<0.01) after comparing non-infected (NI) and *S.* Typhi (ST)-infected targets. The percentage shown within each graph is the estimated sum of all changing clusters. Heatmaps were generated after clustering of concatenated CD8^+^ T_EMRA_ cells after 18 h **(C)** and 40 h **(G)** incubation. Clusters with significant increases are denoted by black outlined rectangles. The varying levels of intensity on the expression of each marker are shown in the blue (low) to red (high) scale. Panels **(D, H)** depict examples of the validation of a cluster showing significant differences between NI (blue) and ST (red) target cells, and the corresponding percentage change (gray), as calculated using a conventional formula: $[\frac{ST-NI}{NI}]*100$. Comparisons between groups were evaluated with Wilcoxon match-pairs signed rank tests, using GraphPad software version 11.0.0. Medians with interquartile ranges are shown in each bar, each dot represents individual data from each participant [panels **(D, H)**]; markers selected for the supervised gating to validate the unsupervised analyses are shown below each graph. **p<0.01, ***p<0.001.

##### 3.2.1.4 CD4^+^ T central memory

CD4^+^ T_CM_ are characterized by proliferation and differentiation to multiple effector subsets with a more restricted TCR repertoire (50, 52, 53). We found that CD4^+^ T_CM_ comprised ~6.48% of CD3^+^ lymphocytes. In this analysis, two out of 17 clusters showed significant increases in cultures with ST-infected target cells after 18 h incubation, highlighting HLADR, CD95, KIR, and CD27 markers distributed in both clusters that represented ~8% of the cluster events in the concatenated file (**Figures 5A–D**; **Supplementary Figure 11**). After 40 h incubation, four out of 17 clusters showed significant increases in cultures with ST-infected targets when compared to NI, also emphasizing markers such as HLADR, CD95, CD27, ICOS, and KIR in all validated clusters, accounting for ~30% of cluster events. (**Figures 5E–H**; **Supplementary Figure 11**).

**Figure 5:**
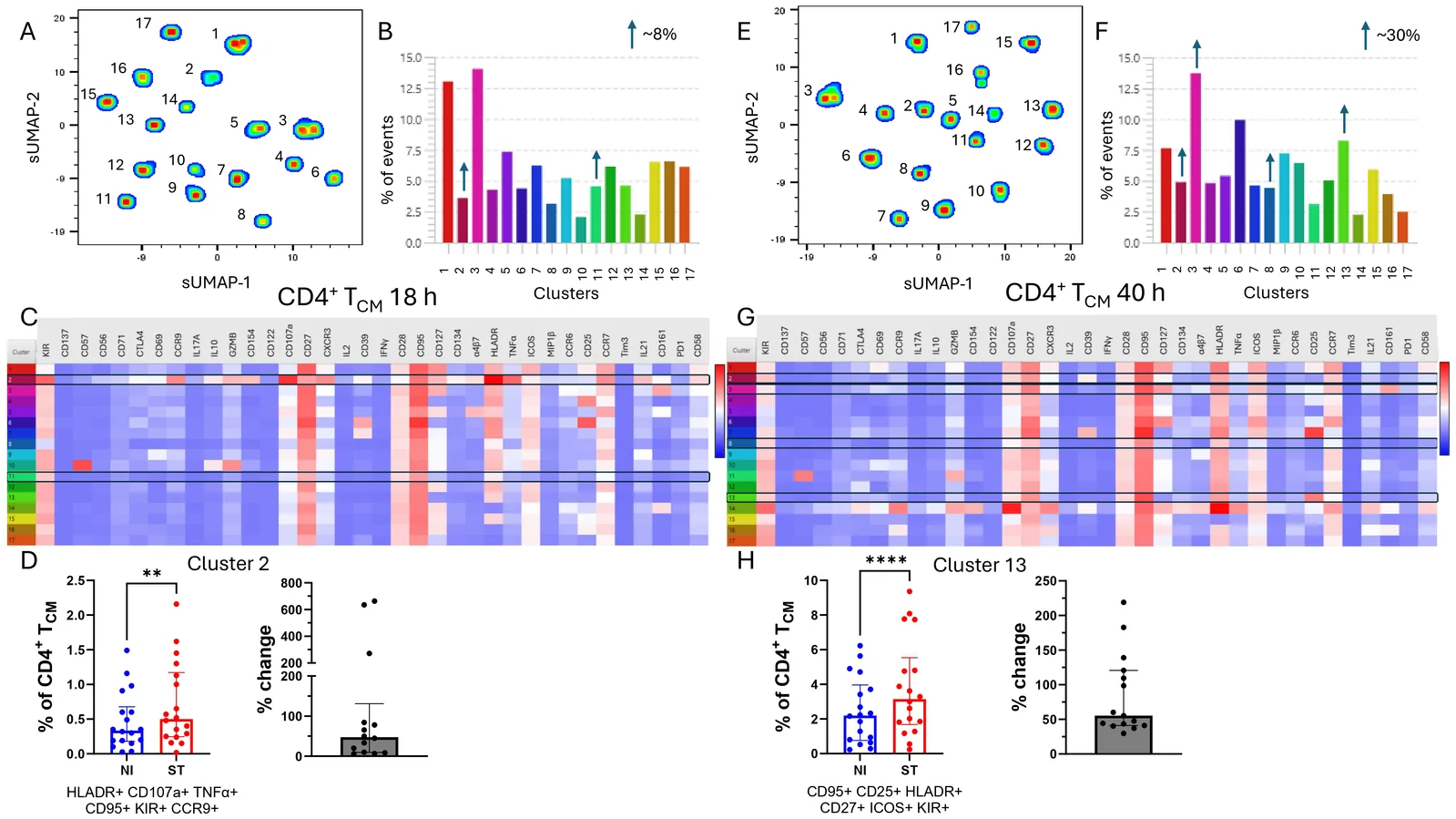
Unsupervised high-dimensional analysis of CD4^+^ T central memory (T_CM_) cells after ex-vivo exposure to *S.* Typhi-infected autologous targets in 18 healthy volunteers. PhenoGraph clusters visualized on UMAP reference maps of CD4^+^ T_CM_ cells after an 18 h **(A)** and 40 h **(E)** PBMC co-culture with autologous EBV-transformed lymphoblastoid B cell lines. Panels **(B, F)** show hierarchical clusterings of concatenated CD4^+^ T_CM_ cells after 18 h **(B)** and 40 h **(F)** incubation, respectively. The arrows show clusters with significant increases (p<0.01) after comparing non-infected (NI) and *S.* Typhi (ST)-infected targets. The percentage shown within each graph is the estimated sum of all changing clusters. Heatmaps were generated after clustering of concatenated CD4^+^ T_CM_ cells after 18 h **(C)** and 40 h **(G)** incubation. Clusters with significant increases are denoted by black outlined rectangles. The varying levels of intensity on the expression of each marker are shown in the blue (low) to red (high) scale. Panels **(D, H)** depict examples of the validation of a cluster showing significant differences between NI (blue) and ST (red) target cells, and the corresponding percentage change (gray), as calculated using a conventional formula: $[\frac{ST-NI}{NI}]*100$. Comparisons between groups were evaluated with Wilcoxon match-pairs signed rank tests, using GraphPad software version 11.0.0. Medians with interquartile ranges are shown in each bar, each dot represents individual data from each participant [panels **(D, H)**]; markers selected for the supervised gating to validate the unsupervised analyses are shown below each graph. **p<0.01, ****p<0.0001.

##### 3.2.1.5 CD4^+^ T effector memory

CD4^+^ T_EM_ are abundant in circulation, where they elicit cytokine-based effector responses to a cognate antigen (50, 52, 53). In this study, CD4^+^ T_EM_ represented ~14.1% of CD3^+^ lymphocytes. In contrast to CD8^+^ T_EM_, which had more clusters with significant increases after 40 h incubation, more CD4^+^ T_EM_ validated clusters showed *S.* Typhi-related increases after 18 h. After 18 h incubation, seven out of 19 clusters showed significant increases in cultures with ST-infected target cells compared to NI, accounting for ~34% of the cluster events in the concatenated file (**Figures 6A–D**; **Supplementary Figure 12**), with the most expressed markers being CD95, CD27, HLADR, CCR7, CD28, and KIR in the validated clusters. After 40 h incubation, two out of 17 clusters showed significant increases in cultures with ST-infected targets when compared to NI, comprising ~8% of cluster events. (**Figures 6E–H**; **Supplementary Figure 12**). The heatmap revealed a high expression of CD95, HLADR, CD25, and ICOS in both validated clusters (**Figure 6G**).

**Figure 6:**
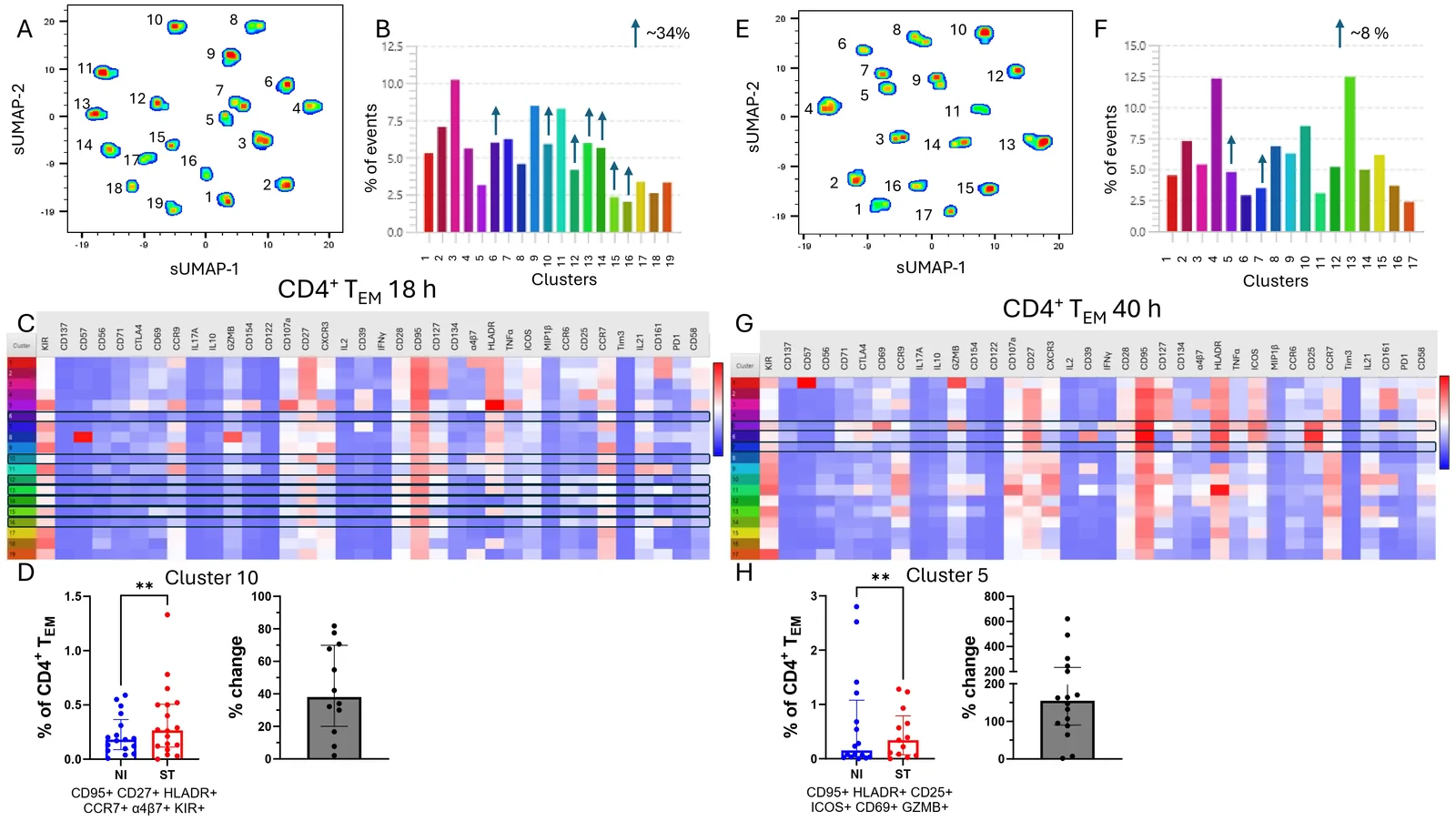
Unsupervised high-dimensional analysis of CD4^+^ T effector memory (T_EM_) cells after ex-vivo exposure to *S.* Typhi-infected autologous targets in 18 healthy volunteers. PhenoGraph clusters visualized on UMAP reference maps of CD4^+^ T_EM_ cells after an 18 h **(A)** and 40 h **(E)** PBMC co-culture with autologous EBV-transformed lymphoblastoid B cell lines. Panels **(B, F)** show hierarchical clusterings of concatenated CD4^+^ T_EM_ cells after 18 h **(B)** and 40 h **(F)** incubation, respectively. The arrows show clusters with significant increases (p<0.01) after comparing non-infected (NI) and *S.* Typhi (ST)-infected targets. The percentage shown within each graph is the estimated sum of all changing clusters. Heatmaps were generated after clustering of concatenated CD4^+^ T_EM_ cells after 18 h **(C)** and 40 h **(G)** incubation. Clusters with significant increases are denoted by black outlined rectangles. The varying levels of intensity on the expression of each marker are shown in the blue (low) to red (high) scale. Panels **(D, H)** depict examples of the validation of a cluster showing significant differences between NI (blue) and ST (red) target cells, and the corresponding percentage change (gray), as calculated using a conventional formula: $[\frac{ST-NI}{NI}]*100$. Comparisons between groups were evaluated with Wilcoxon match-pairs signed rank tests, using GraphPad software version 11.0.0. Medians with interquartile ranges are shown in each bar, each dot represents individual data from each participant [panels **(D, H)**]; markers selected for the supervised gating to validate the unsupervised analyses are shown below each graph. **p<0.01.

##### 3.2.1.6 CD4^+^ T effector memory re-expressing CD45RA

CD4^+^ T_EMRA_, which are less abundant than the CD8^+^ T_EMRA_ subset but with similar differentiation characteristics, represent a highly effector population following repeated antigenic stimulation (50, 53, 54). We found that CD4^+^ T_EMRA_ comprised ~4.48% of CD3^+^ lymphocytes. In this analysis, one cluster showed significant increases in cultures with ST-infected target cells after 18 h incubation, accounting for ~18% of cluster events in the concatenated file (**Figures 7A–D**), and two clusters after 40 h, representing ~16% of cluster events (**Figures 7E–H**). Interestingly, at 18 h, the cluster was validated using several homing markers (CCR7, CCR9, and CXCR3). However, at 40 h, the clusters were validated with more activation than homing markers (CD95, HLADR, CD27, and CCR7).

**Figure 7:**
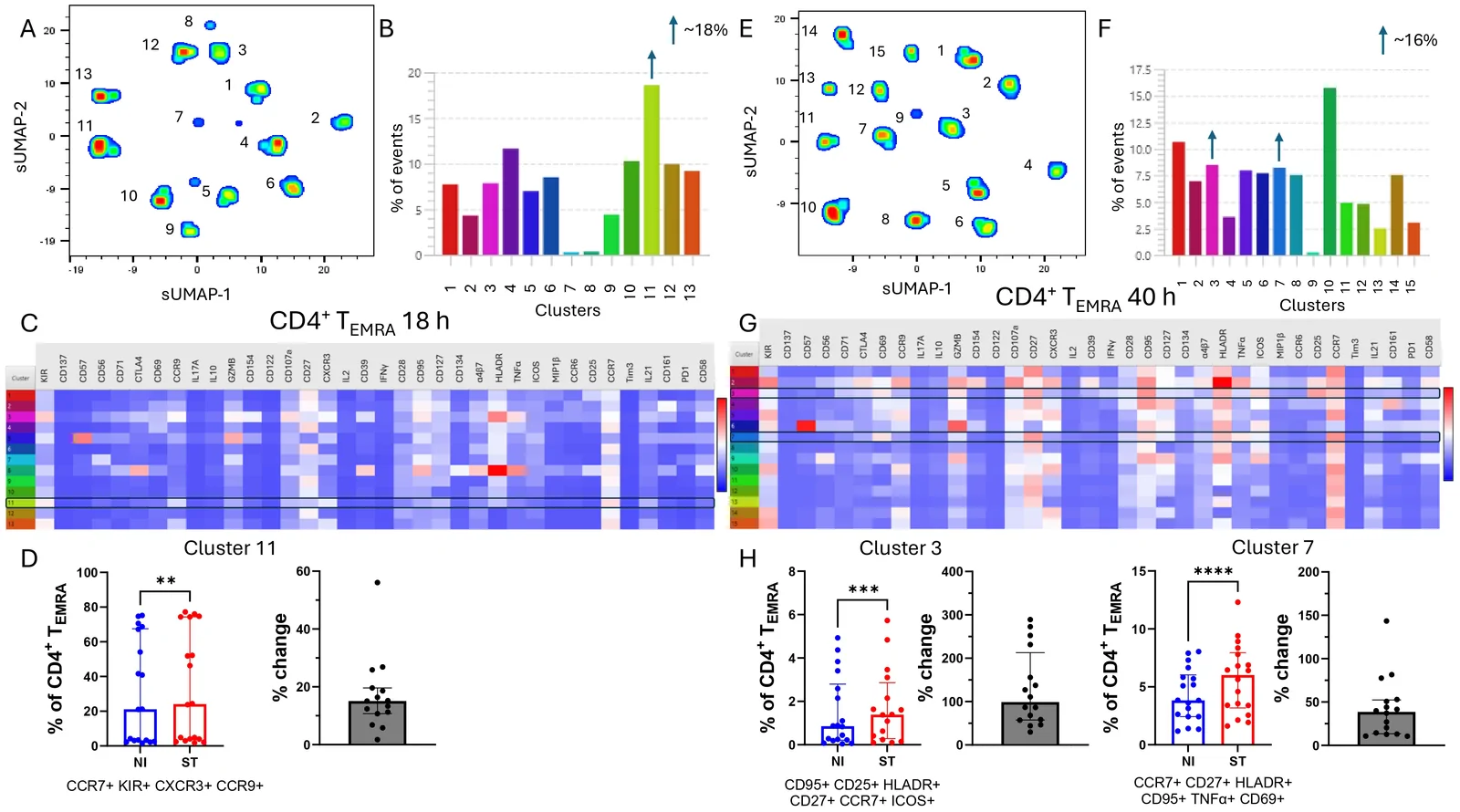
Unsupervised high-dimensional analysis of terminally differentiated CD4^+^ T effector memory cells re-expressing CD45RA (T_EMRA_) after ex-vivo exposure to *S.* Typhi-infected autologous targets in 18 healthy volunteers. PhenoGraph clusters visualized on UMAP reference maps of CD4^+^ T_EMRA_ cells after 18 h **(A)** and 40 h **(E)** incubation. Panels **(B, F)** show hierarchical clusterings of concatenated CD4^+^ T_EMRA_ cells after 18 h **(B)** and 40 h **(F)** incubation, respectively. The arrows show clusters with significant increases (p<0.01) after comparing non-infected (NI) and *S.* Typhi (ST)-infected targets. The percentage shown within each graph is the estimated sum of all changing clusters. Heatmaps were generated after clustering of concatenated CD4^+^ T_EMRA_ cells after 18 h **(C)** and 40 h **(G)** incubation. Clusters with significant increases are denoted by black outlined rectangles. The varying levels of intensity on the expression of each marker are shown in the blue (low) to red (high) scale. Panels **(D, H)** depict the validation of the clusters showing significant differences between NI (blue) and ST (red) target cells, and the corresponding percentage change (gray), as calculated using a conventional formula: [*(ST-NI)/NI*]∗100. Comparisons between groups were evaluated with Wilcoxon match-pairs signed rank tests, using GraphPad software version 11.0.0. Medians with interquartile ranges are shown in each bar, each dot represents individual data from each participant [panels **(D, H)**]; markers selected for the supervised gating to validate the unsupervised analyses are shown below each graph. **p<0.01, ***p<0.001, ****p<0.0001.

##### 3.2.1.7 Circulating CD4^+^ T follicular helper cells

Another important circulating population that facilitates B memory and plasma cell differentiation, similar to T follicular helper cells residing in germinal centers, is the CD4^+^ cT_FH_ subset, characterized by CXCR5 expression (55). These cells are typically divided into Th1, Th2, and Th17-like phenotypes, based on CXCR3 and CCR6 expression (21, 27, 29, 36). We found that CD4^+^ cT_FH_ comprised ~3.12% of CD3^+^ lymphocytes. In this analysis, two out of 11 clusters showed significant decreases in cultures with ST-infected target cells after 18 h incubation, highlighting CD27, CD62L, CCR7, CD95, CD28, and KIR markers distributed in both clusters that represented ~13% of cluster events in the concatenated file (**Figures 8A–D**). According to the generated heatmap, cluster 3 can be defined as cT_FH_1 (CXCR3^+^CCR6^−^), and cluster 8 as cT_FH_2 (CXCR3^−^CCR6^−^) (**Figures 8A, C**). In contrast, after 40 h incubation, two out of 13 clusters showed significant increases in cultures with ST-infected targets when compared to NI, also emphasizing CD27, CD62L, and CD95 markers, together with HLADR, ICOS, and CD25 in both validated clusters (**Figures 8E–H**). Both clusters were defined as cT_FH_1 (CXCR3^+^CCR6^−^; **Figures 8E, G**) and accounted for ~10% of cluster events.

**Figure 8:**
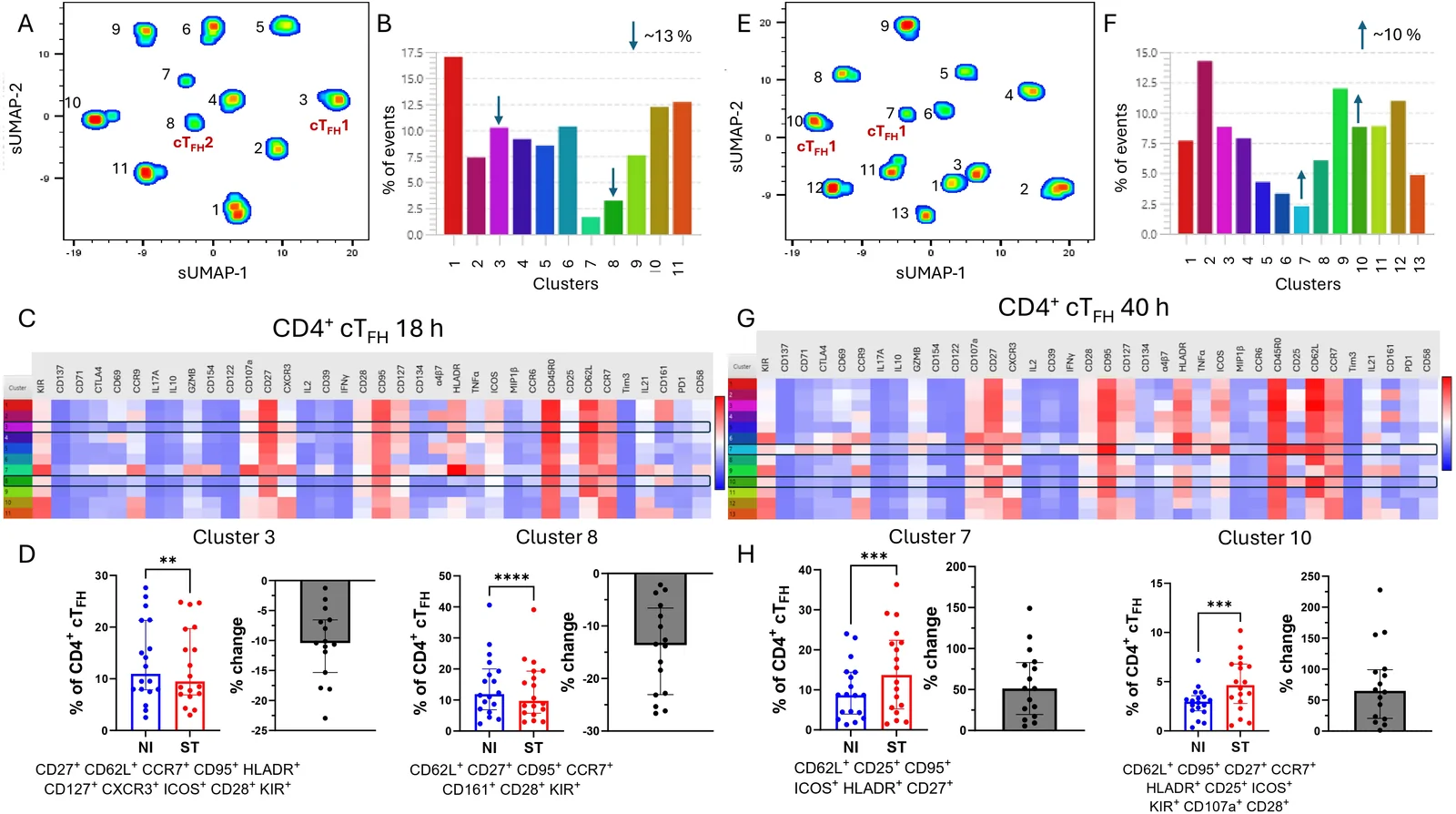
Unsupervised high-dimensional analysis of CD4^+^ circulating T follicular helper (cT_FH_) cells after ex-vivo exposure to *S.* Typhi-infected autologous targets in 18 healthy volunteers. PhenoGraph clusters visualized on UMAP reference maps of CD4^+^ cT_FH_ cells after an 18 h **(A)** and 40 h **(E)** PBMC co-culture with autologous EBV-transformed lymphoblastoid B cell lines. Panels **(B, F)** show hierarchical clusterings of concatenated CD4^+^ cT_FH_ cells after 18 h **(B)** and 40 h **(F)** incubation, respectively. The arrows show clusters with significant increases or decreases (p<0.01) after comparing non-infected (NI) and *S.* Typhi (ST)-infected targets. The percentage shown within each graph is the estimated sum of all changing clusters. Heatmaps were generated after clustering of concatenated CD4^+^ cT_FH_ cells after 18 h **(C)** and 40 h **(G)** incubation. Clusters with significant increases are denoted by black outlined rectangles. The varying levels of intensity on the expression of each marker are shown in the blue (low) to red (high) scale. Panels **(D, H)** depict the validation of the clusters showing significant differences between NI (blue) and ST (red) target cells, and the corresponding percentage change (gray), as calculated using a conventional formula: $[\frac{ST-NI}{NI}]*100$. Comparisons between groups were evaluated with Wilcoxon match-pairs signed rank tests, using GraphPad software version 11.0.0. Medians with interquartile ranges are shown in each bar, each dot represents individual data from each participant [panels **(D, H)**]; markers selected for the supervised gating to validate the unsupervised analyses are shown below each graph. **p<0.01, ***p<0.001, ****p<0.0001.

##### 3.2.1.8 T stem cell memory

T_SCM_ constitute a memory cell subset with stem cell properties, with a mixed naïve-memory phenotype that suggests longevity and high plasticity, both in CD4^+^ and CD8^+^ subsets (52, 56–58). We used a very stringent 10-marker gating strategy to clearly define this subset and distinguish it from other memory populations (**Figure 3**). We found that CD4^+^ T_SCM_ comprised ~11% of CD3^+^ lymphocytes, while CD8^+^ T_SCM_ represented ~3.39%. In the CD4^+^ T_SCM_ analysis after 18 h incubation, four out of 14 clusters showed significant increases in cultures with ST-infected target cells compared to NI, accounting for ~33% of the cluster events in the concatenated file (**Figures 9A–D**; **Supplementary Figure 13E**), with the most expressed markers being HLADR, KIR, and CD107a in the validated clusters. Moreover, after 40 h incubation, one out of 15 clusters showed a significant increase in cultures with ST-infected targets when compared to NI, comprising ~8% of cluster events (**Supplementary Figures 13A–D**). The heatmap revealed a high expression of CD95, HLADR, KIR, and ICOS in the validated cluster (**Supplementary Figure 13C**). In the CD8^+^ T_SCM_ analysis after 18 h incubation, no cluster showed significant differences between cultures with ST-infected target cells and NI (**Supplementary Figures 14A–C**). However, after 40 h incubation, four out of 15 clusters showed significant increases in cultures with ST-infected targets when compared to NI, (**Figures 9E–H**; **Supplementary Figure 14D**), with the most expressed markers being HLADR, CD107a, and KIR, and accounting for ~18% of cluster events in the concatenated file. Interestingly, HLADR and integrin α4β7 were the main markers expressed in cluster 10, which showed a significant decrease in cultures with ST-infected targets when compared to NI, representing ~3% of cluster events (**Figures 9E–H**; **Supplementary Figure 14D**).

**Figure 9:**
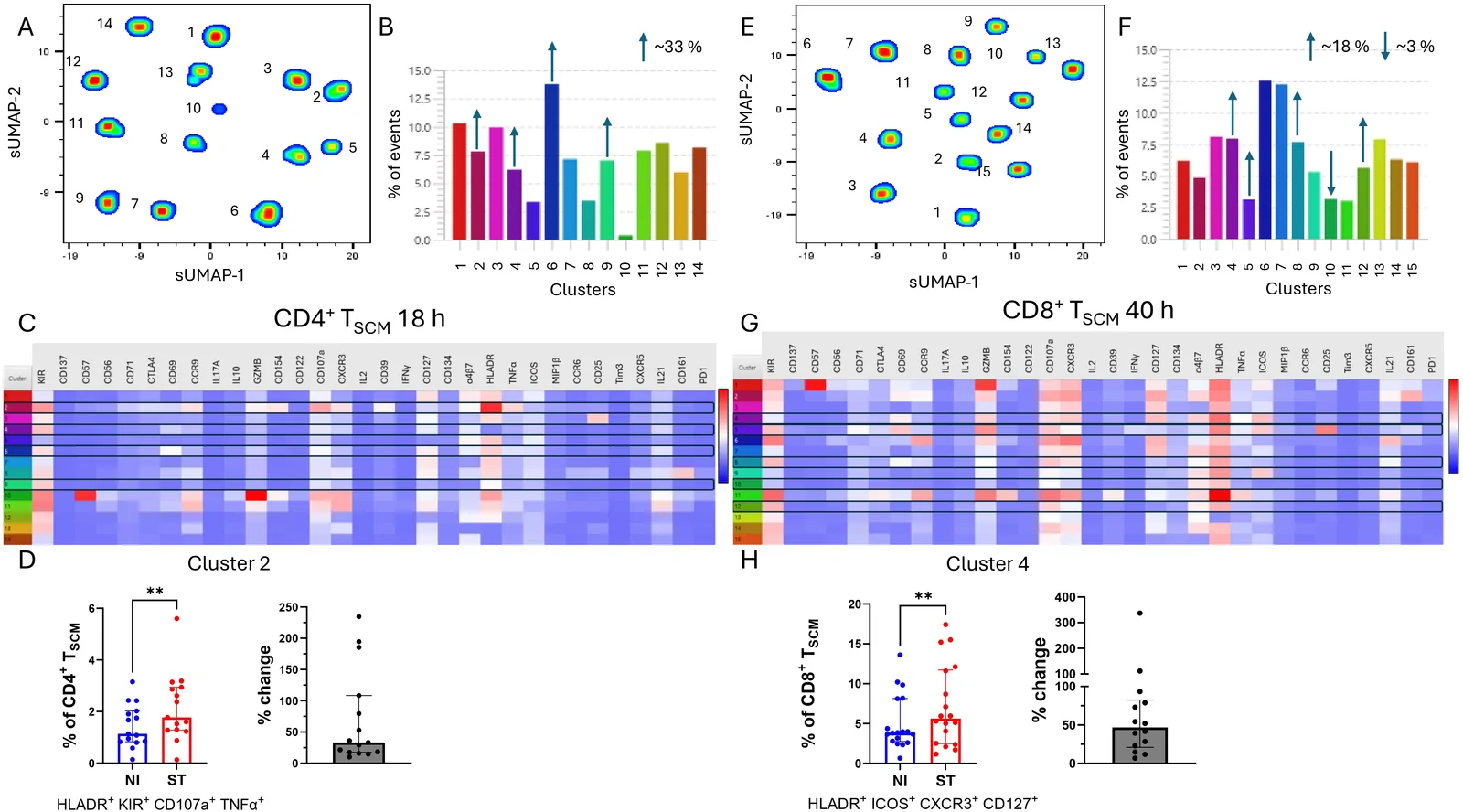
Unsupervised high-dimensional analysis of T stem cell memory (T_SCM_) cell subsets after ex-vivo exposure to *S.* Typhi-infected autologous targets in 18 healthy volunteers. PhenoGraph clusters visualized on UMAP reference maps of CD4^+^ T_SCM_ **(A)** or CD8^+^ T_SCM_ **(E)** cells after an 18 h **(A)** or 40 h **(E)** PBMC co-culture with autologous EBV-transformed lymphoblastoid B cell lines. Panels **(B, F)** show hierarchical clusterings of concatenated CD4^+^ T_SCM_ **(B)** or CD8^+^ T_SCM_ **(F)** cells after 18 h or 40 h incubation, respectively. The arrows show clusters with significant increases or decreases (p<0.01) after comparing non-infected (NI) and *S.* Typhi (ST)-infected targets. The percentage shown within each graph is the estimated sum of all changing clusters. Heatmaps were generated after clustering of concatenated CD4^+^ T_SCM_ **(C)** or CD8^+^ T_SCM_ **(G)** cells after 18 h **(C)** or 40 h **(G)** incubation. Clusters with significant increases are denoted by black outlined rectangles. The varying levels of intensity on the expression of each marker are shown in the blue (low) to red (high) scale. Panels **(D, H)** depict examples of the validation of a cluster showing significant differences between NI (blue) and ST (red) target cells, and the corresponding percentage change (gray), as calculated using a conventional formula: $[\frac{ST-NI}{NI}]*100$. Comparisons between groups were evaluated with Wilcoxon match-pairs signed rank tests, using GraphPad software version 11.0.0. Medians with interquartile ranges are shown in each bar, each dot represents individual data from each participant [panels **(D, H)**]; markers selected for the supervised gating to validate the unsupervised analyses are shown below each graph. **p<0.01.

##### 3.2.1.9 T regulatory cells

CD4^+^ T regulatory cells (T_reg_) play an important role in immune tolerance by controlling other T and B cell activation through direct cell-to-cell interactions and suppressive cytokine production. After antigenic exposure, the persistence of these cells might limit tissue damage and chronic inflammation (59). We found that CD4^+^ T_reg_ represented ~2.78% of CD3^+^ lymphocytes. In this analysis, one out of 15 clusters showed a significant increase in cultures with ST-infected target cells after 18 h incubation, representing ~12% of cluster events in the concatenated file, which was validated with several activation markers, such as CD95, HLADR, CD27, CD39, KIR, and ICOS (**Figures 10A–C**; **Supplementary Figure 15A**). Of note, two clusters showed significant decreases in cultures with ST-infected target cells when compared to NI, highlighting CD62L, CD27, and CD95 markers in both clusters that accounted for ~13% of cluster events (**Figures 10A, B**; **Supplementary Figures 15A, B**). After 40 h incubation, one out of 13 clusters showed a significant increase in cultures with ST-infected targets, also emphasizing markers such as CD95, HLADR, CD62L, CD27, ICOS, CD161, and KIR, comprising ~10% of cluster events in the concatenated file (**Figures 10D–F**; **Supplementary Figure 15C**). In contrast, cluster 1 showed a significant decrease in cultures with ST-infected targets when compared to NI, validated with CD95, HLADR, CD62L, CD27, ICOS, CD39, and CD134 as the most expressed markers (**Figures 10D–F**; **Supplementary Figures 15C, D**), also accounting for ~10% of cluster events.

**Figure 10:**
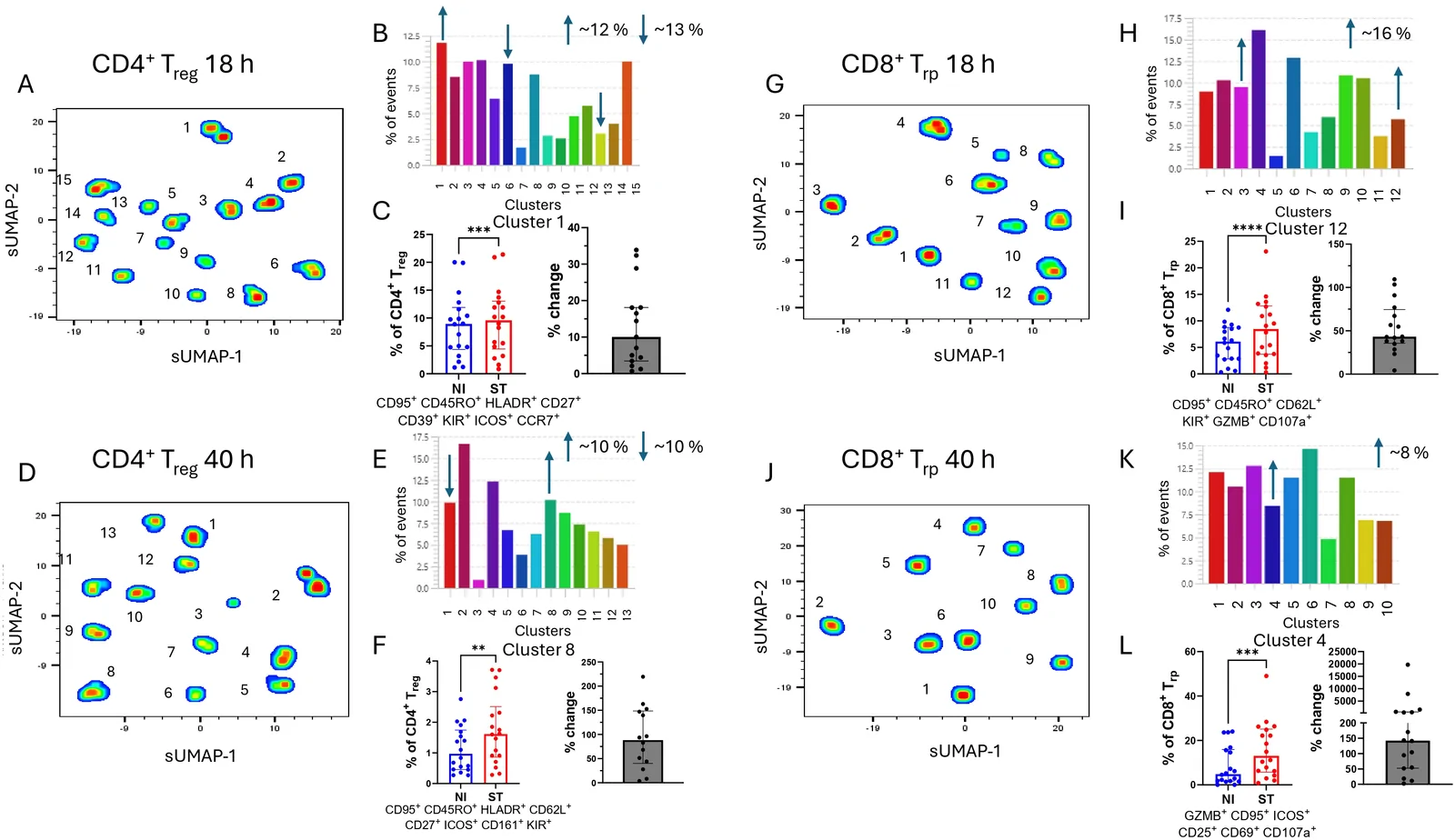
Unsupervised high-dimensional analysis of CD4^+^ T regulatory (T_reg_) and CD8^+^ T regulatory precursor (T_rp_) cell subsets after ex-vivo exposure to *S.* Typhi-infected autologous targets in 18 healthy volunteers. PhenoGraph clusters visualized on UMAP reference maps of CD4^+^ T_reg_ **(A, D)** or CD8^+^ T_rp_ **(G, J)** cells after an 18 h **(A, G)** and 40 h **(D, J)** PBMC co-culture with autologous EBV-transformed lymphoblastoid B cell lines. Panels **(B, E, H, K)** show hierarchical clusterings of concatenated CD4^+^ T_reg_ **(B, E)** or CD8^+^ T_rp_ **(H, K)** cells after 18 h **(B, H)** and 40 h **(E, K)** incubation. The arrows show clusters with significant increases or decreases (p<0.01) after comparing non-infected (NI) and *S.* Typhi (ST)-infected targets. The percentage shown within each graph is the estimated sum of all changing clusters. Heatmaps were generated after clustering of concatenated CD4^+^ T_reg_ or CD8^+^ T_rp_ cells after 18 h and 40 h incubation (see **Supplementary Figures 15**, **16**), followed by supervised validation of clusters showing significant differences between NI and ST target cells, from which examples are depicted in panels **(C, F, I, L)**. Comparisons between NI (blue) and ST (red) target cells were evaluated with Wilcoxon match-pairs signed rank tests, using GraphPad software version 11.0.0. The percentage change (gray) was calculated using a conventional formula: $[\frac{ST-NI}{NI}]*100$. Medians with interquartile ranges are shown in each bar, each dot represents individual data from each participant [panels **(C, F, I, L)**]; markers selected for the supervised gating to validate the unsupervised analyses are shown below each graph. **p<0.01, ***p<0.001, ****p<0.0001.

CD8^+^ T regulatory precursors (T_rp_) are early-phenotype suppressive cells before their differentiation to a subset that modulates excessive cytotoxic and inflammatory responses (60). We found that CD8^+^ T_rp_ represented ~1.33% of CD3^+^ lymphocytes. In this analysis, two out of 12 clusters showed significant increases in cultures with ST-infected target cells after 18 h incubation (**Figures 10G–I**; **Supplementary Figure 16A, B**) and one cluster after 40 h (**Figures 10J–L**; **Supplementary Figure 16C**), comprising ~16% and ~8% of cluster events in the concatenated file, respectively. At 18 h, both clusters were validated using CD107a, KIR, and CD95 as the most expressed markers (**Figure 10I**; **Supplementary Figure 16A, B**); at 40 h, cluster 4 was validated with more activation markers, including CD95, ICOS, CD25, and CD69 (**Figure 10L**; **Supplementary Figure 16C**).

#### 3.2.2 Non-conventional T cell subsets

##### 3.2.2.1 Mucosal-associated invariant T cells

CD8^+^ MAIT cells are invariant lymphocytes that recognize riboflavin metabolites from pathogens (bacteria and fungi), eliciting rapid cytokine responses. MAIT cells are mostly found in mucosal sites and peripheral blood, where they modulate local inflammatory responses (61). These cells express a semi-conserved TCR (human Vα7.2-Jα33) and CD161 (62); the latter has been shown to be downregulated during *S.* Typhi infection (10). We found that CD8^+^ MAIT represent ~0.19% of CD3^+^ lymphocytes. In this analysis, four out of 11 clusters showed significant increases in cultures with ST-infected target cells after 18 h incubation, highlighting CD107a and CD45RO markers in all validated clusters that accounted for ~38% of cluster events in the concatenated file (**Figures 11A–D**; **Supplementary Figure 17A**). After 40 h incubation, seven out of 12 clusters showed significant increases in cultures with ST-infected targets when compared to NI, comprising ~61% of cluster events and also emphasizing KIR and CD45RO markers in some of the validated clusters, together with other activation markers such as CD25, CD69, ICOS, CD95, and HLADR. These clusters also expressed cytokines (IFNγ) and other cytotoxicity markers (GZMB and CD107a). Of note, clusters 4 and 12 showed significant decreases in cultures with ST-infected target cells compared to NI, emphasizing the expression of CD45RO, CD95, and CD127 markers in both clusters, representing ~9% of cluster events (**Figures 11E–H**; **Supplementary Figure 17B**). Interestingly, the percentages of change in the presence of ST-infected target cells versus NI targets were the strongest among all evaluated cell subsets (**Figure 11**; **Supplementary Figure 17**).

**Figure 11:**
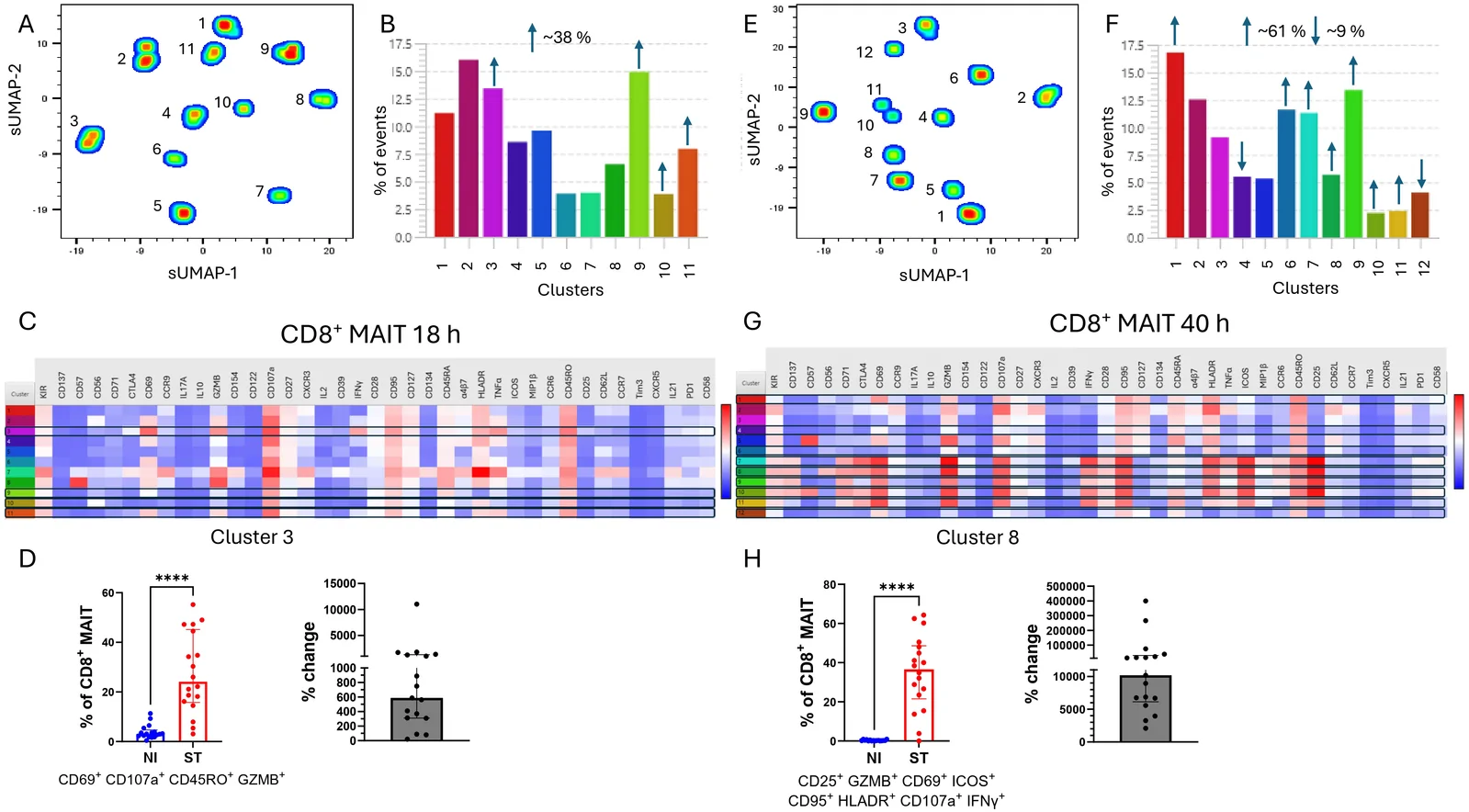
Unsupervised high-dimensional analysis of CD8^+^ mucosal associated invariant T (MAIT) cells after ex-vivo exposure to *S.* Typhi-infected autologous targets in healthy volunteers. PhenoGraph clusters visualized on UMAP reference maps of CD8^+^ MAIT cells after an 18 h (n=15) **(A)** and 40 h (n=14) **(E)** PBMC co-culture with autologous EBV-transformed lymphoblastoid B cell lines. Panels **(B, F)** show hierarchical clusterings of concatenated CD8^+^ MAIT cells after 18 h **(B)** and 40 h **(F)** incubation, respectively. The arrows show clusters with significant increases or decreases (p<0.01) after comparing non-infected (NI) and *S.* Typhi (ST)-infected targets. The percentage shown within each graph is the estimated sum of all changing clusters. Heatmaps were generated after clustering of concatenated CD8^+^ MAIT cells after 18 h **(C)** and 40 h **(G)** incubation. Clusters with significant increases are denoted by black outlined rectangles. The varying levels of intensity on the expression of each marker are shown in the blue (low) to red (high) scale. Panels **(D, H)** depict examples of the validation of a cluster showing significant differences between NI (blue) and ST (red) target cells, and the corresponding percentage change (gray), as calculated using a conventional formula: $[\frac{ST-NI}{NI}]*100$. Comparisons between groups were evaluated with Wilcoxon match-pairs signed rank tests, using GraphPad software version 11.0.0. Medians with interquartile ranges are shown in each bar, each dot represents individual data from each participant [panels **(D, H)**]; markers selected for the supervised gating to validate the unsupervised analyses are shown below each graph. ****p<0.0001.

##### 3.2.2.2 Gamma delta T cells

Tγδ cells are lymphocytes with a restricted TCR repertoire that can be found in circulation and in several tissues (epithelia), where they combine innate and adaptive responses to phosphoantigens and other non-conventional ligands (63). In this study, Tγδ comprised ~1.07% of CD3^+^ lymphocytes. After 18 h incubation, two out of 12 clusters showed significant increases in cultures with ST-infected target cells compared to NI (**Figures 12A–D**; **Supplementary Figure 18A**), with the most expressed markers being HLADR, CD107a, CD95, CXCR3, and GZMB in both clusters that represented ~10% of cluster events in the concatenated file. Moreover, after 40 h incubation, seven out of 12 clusters showed significant increases in cultures with ST-infected targets when compared to NI, accounting for ~58% of the cluster events (**Figures 12E–H**; **Supplementary Figure 18B**). The heatmap revealed a high expression of CD95, GZMB, CD25, and CD107a in most of the validated clusters (**Figure 12G**), and remarkably, several homing markers (CXCR3, CCR9, and CD62L) were highly expressed in some of the validated clusters.

**Figure 12:**
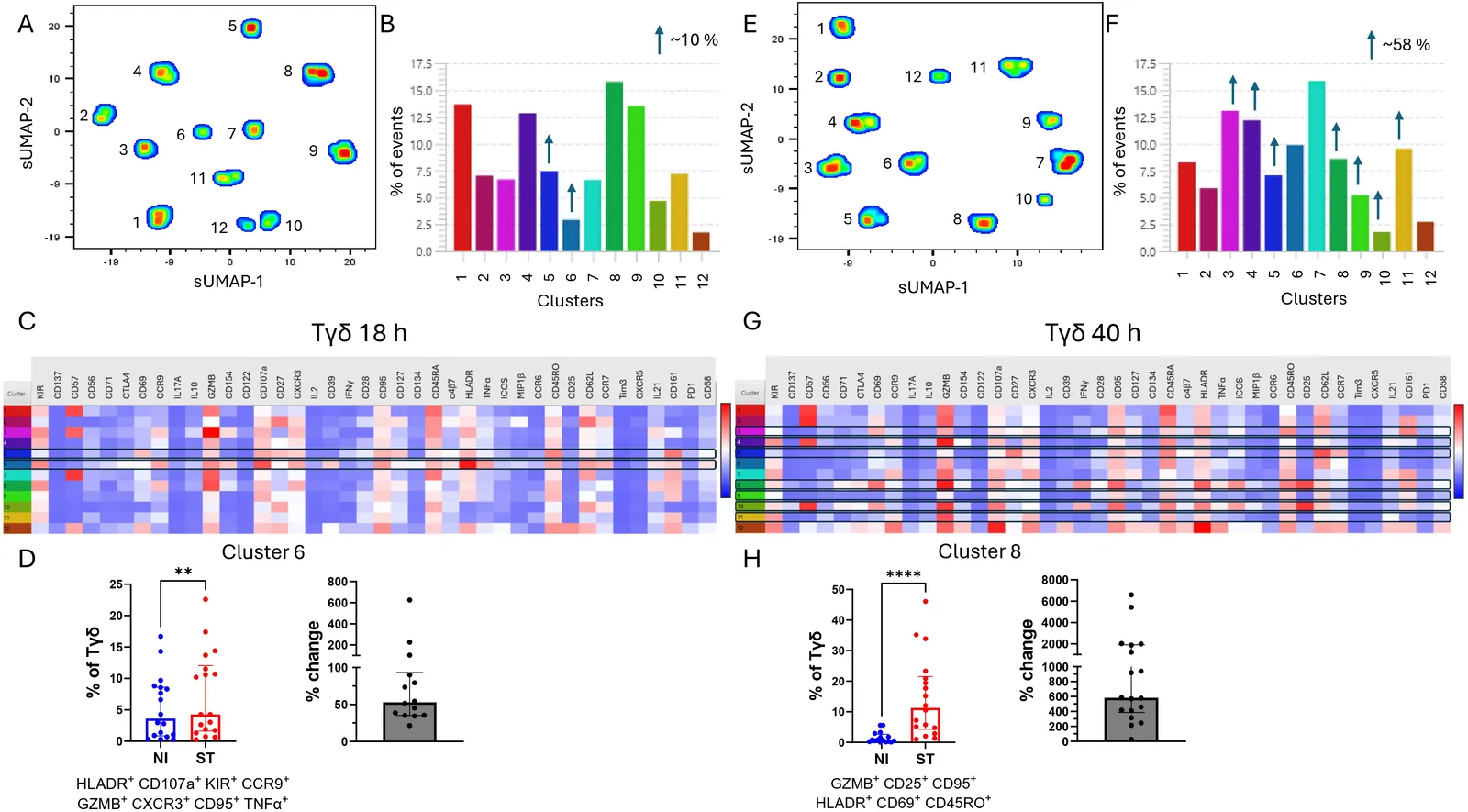
Unsupervised high-dimensional analysis of T gamma-delta (Tγδ) cells after ex-vivo exposure to *S.* Typhi-infected autologous targets in 18 healthy volunteers. PhenoGraph clusters visualized on UMAP reference maps of Tγδ cells after an 18 h **(A)** and 40 h **(E)** PBMC co-culture with autologous EBV-transformed lymphoblastoid B cell lines. Panels **(B, F)** show hierarchical clusterings of concatenated Tγδ cells after 18 h **(B)** and 40 h **(F)** incubation, respectively. The arrows show clusters with significant increases (p<0.01) after comparing non-infected (NI) and *S.* Typhi (ST)-infected targets. The percentage shown within each graph is the estimated sum of all changing clusters. Heatmaps were generated after clustering of concatenated Tγδ cells after 18 h **(C)** and 40 h **(G)** incubation. Clusters with significant increases are denoted by black outlined rectangles. The varying levels of intensity on the expression of each marker are shown in the blue (low) to red (high) scale. Panels **(D, H)** depict examples of the validation of a cluster showing significant differences between NI (blue) and ST (red) target cells, and the corresponding percentage change (gray), as calculated using a conventional formula: $[\frac{ST-NI}{NI}]*100$. Comparisons between groups were evaluated with Wilcoxon match-pairs signed rank tests, using GraphPad software version 11.0.0. Medians with interquartile ranges are shown in each bar, each dot represents individual data from each participant [panels **(D, H)**]; markers selected for the supervised gating to validate the unsupervised analyses are shown below each graph. **p<0.01, ****p<0.0001.

##### 3.2.2.3 Natural-killer T cells

NKT cells share characteristics of both Natural-Killer and T cells; they recognize lipids and glycolipids through a semi-conserved TCR, eliciting ample cytokine and cytotoxic functions to modulate innate and adaptive responses (64). We found that NKT represent ~0.91% of CD3^+^ lymphocytes. In this analysis, five out of 15 clusters showed significant increases in cultures with ST-infected target cells after 18 h incubation, highlighting CD95, KIR, CD107a, GZMB, and CD45RA markers in most of the validated clusters, which accounted for ~46% of cluster events in the concatenated file (**Figures 13A–D**; **Supplementary Figure 19A**). After 40 h incubation, five out of 14 clusters showed significant increases in cultures with ST-infected targets when compared to NI, comprising ~36% of cluster events and also emphasizing CD95, KIR, CD107a, and GZMB in most of the validated clusters, together with other activation markers such as CD25 and HLADR. Most of these clusters also expressed homing markers (CCR9, CCR7, CXCR3, CD62L, and integrin α4β7) (**Figures 13E–H**; **Supplementary Figure 19B**).

**Figure 13:**
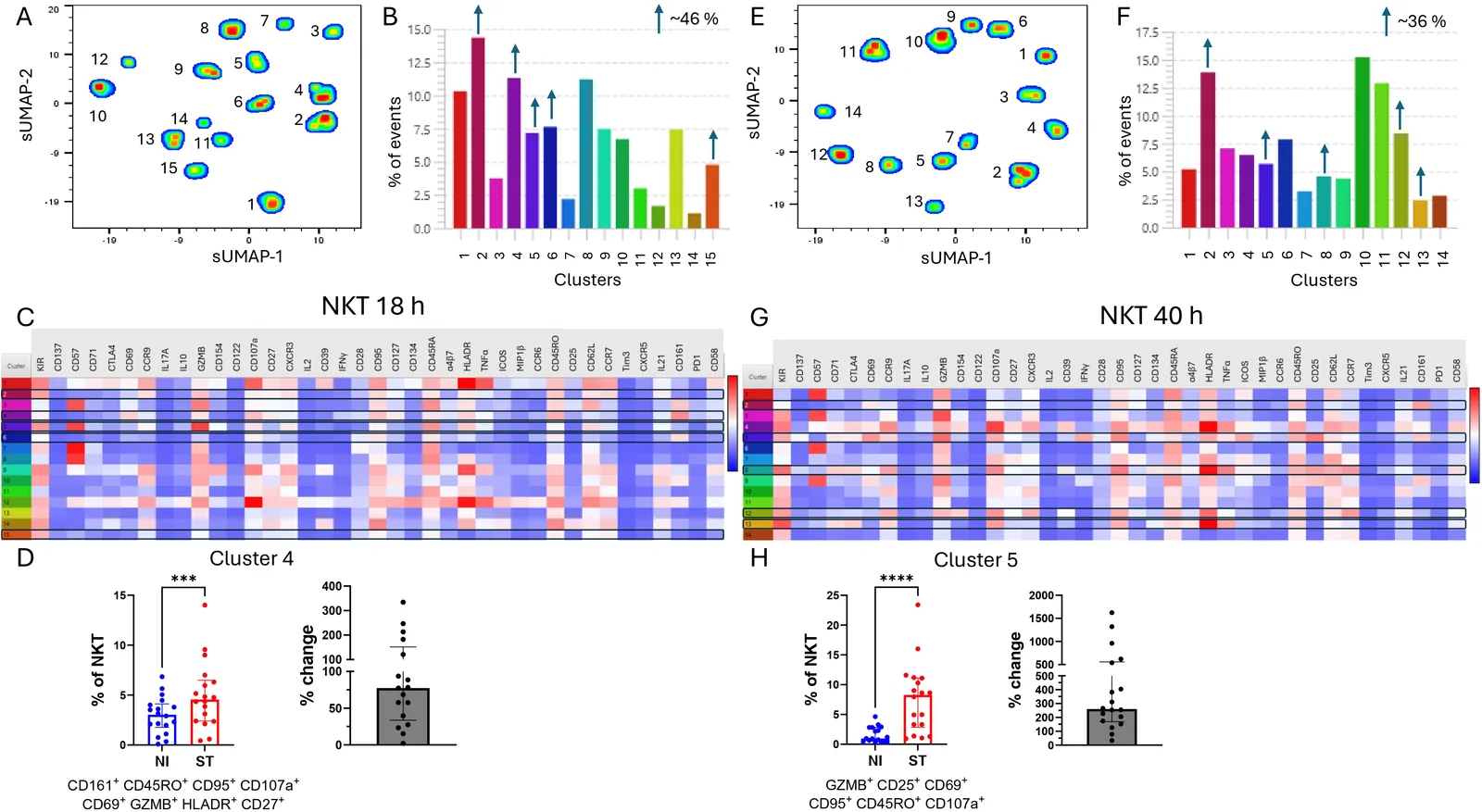
Unsupervised high-dimensional analysis of Natural-Killer T (NKT) cells after ex-vivo exposure to *S.* Typhi-infected autologous targets in 18 healthy volunteers. PhenoGraph clusters visualized on UMAP reference maps of NKT cells after an 18 h **(A)** and 40 h **(E)** PBMC co-culture with autologous EBV-transformed lymphoblastoid B cell lines. Panels **(B, F)** show hierarchical clustering of concatenated NKT cells after 18 h **(B)** and 40 h **(F)** incubation, respectively. The arrows show clusters with significant increases (p<0.01) after comparing non-infected (NI) and *S.* Typhi (ST)-infected targets. The percentage shown within each graph is the estimated sum of all changing clusters. Heatmaps were generated after clustering of concatenated NKT cells after 18 h **(C)** and 40 h **(G)** incubation. Clusters with significant increases are denoted by black outlined rectangles. The varying levels of intensity on the expression of each marker are shown in the blue (low) to red (high) scale. Panels **(D, H)** depict examples of the validation of a cluster showing significant differences between NI (blue) and ST (red) target cells, and the corresponding percentage change (gray), as calculated using a conventional formula: $[\frac{ST-NI}{NI}]*100$. Comparisons between groups were evaluated with Wilcoxon match-pairs signed rank tests, using GraphPad software version 11.0.0. Medians with interquartile ranges are shown in each bar, each dot represents individual data from each participant [panels **(D, H)**]; markers selected for the supervised gating to validate the unsupervised analyses are shown below each graph. ***p<0.001, ****p<0.0001.

### 3.3 Interaction between clusters with significant changes and cytokines/chemokines in culture supernatants

The Spearman correlation matrix was constructed with 39 clusters showing significant responses (p<0.01) from the 18 h incubation analyses and 55 clusters from the 40 h incubation analyses described above, as well as the concentrations of 13 cytokines/chemokines (IL1β, IL2, IL6, IL8, IL10, IL12p70, IL17A, IL18, IL23, IL33, IFNγ, TNFα, and CCL2) measured in the cell culture supernatants collected ~27 h after initiation of the cultures. To enhance readability and facilitate the interpretation of these analyses, we distributed the clusters in two ways: first, the correlation between the 18 h incubation clusters and the cytokines measured in the cell culture supernatants (**Supplementary Figure 20**), as well as the correlation between the 40 h clusters and the cytokines (**Supplementary Figure 20**). Moreover, we divided the selected clusters according to the cell type, e.g. conventional CD4^+^ T cell subsets, conventional CD8^+^ T cell subsets, and non-conventional T cell subsets (MAIT, Tγδ, and NKT), along with the cytokine concentrations in the supernatants (**Supplementary Figures 21A–C**, respectively). To enhance readability, the clusters that did not show any significant p value (p<0.01) were not included in the heatmap. The complete matrix is shown in **Supplementary Figure 22**.

Significant correlations were observed in clusters from the same cell subset and the same incubation time (e.g., CD8TEM-cluster 8 positively correlated with CD8TEM-cluster 9 and CD8TEM-cluster 10 at 18 h), but also across cell subsets at the same incubation time (e.g. CD8TEM-cluster 8 positively correlated with CD4TEM-cluster 12 at 18 h, CD4TSCM-cluster2 positively correlated with MAIT-cluster 9 and NKT-cluster 4 at 18 h, NKT-cluster 5 positively correlated with Tγδ-cluster 8 at 40 h, CD4TSCM-cluster 3 positively correlated with CD8TSCM-cluster 4 at 40 h, CD8TSCM-cluster 5 positively correlated with CD8TCM-cluster 3 and CD8TCM-cluster 7 at 40 h) (**Supplementary Figures 20A, B**). Fewer correlations were found between the same cell subset at different incubation times (e.g., cTFH-cluster 3 at 18 h positively correlated with cTFH-cluster 7 at 40 h, cTFH-cluster 8 at 18 h positively correlated with cTFH-cluster 10 at 40 h, MAIT-cluster 10 at 18 h negatively correlated with MAIT-cluster 7 and MAIT-cluster 8 at 40 h), or distinct cell subsets at different incubation times (e.g., NKT-cluster 2 at 18 h negatively correlated with Tγδ -cluster 9 at 40 h, NKT-cluster 4 at 18 h negatively correlated with MAIT-cluster 7 and MAIT-cluster 9 at 40 h) (**Supplementary Figures 21A–C**). Correlations among cytokines were abundant and compelling (e.g., TNFα positively correlated with IL10, IL1β, and IL6, but negatively correlated with IL33; IL17 positively correlated with IL23). However, scarce correlations were significant between cytokines and a validated cluster at 18 h or 40 h incubation. For example, at 18 h incubation, IL17A and IL33 negatively correlated with CD8Trp-cluster 3, CCL2 positively correlated with MAIT-cluster 10, and IL2 positively correlated with CD8TEMRA-cluster 2 (**Supplementary Figure 20**). At 40 h incubation, IL33 positively correlated with CD8TEM-cluster14, IL8 positively correlated with MAIT-cluster 9, and IL12p70 and IL23 positively correlated with NKT-cluster 13 (**Supplementary Figure 20B**). Taken together, these correlation studies between significantly different clusters from the 18 h analyses and the measured cytokines 27 h after initiation of the cultures suggest that these proinflammatory mediators were produced by one or more cell types after stimulation with ST-infected target cells. Interestingly, correlations between cytokines and significant clusters from the 40 h analyses suggest a possible enhancement of cell activation by the cytokine milieu present in cell cultures, induced after stimulation with ST-infected target cells. Moreover, correlations between clusters in distinct cell subsets suggest that T-CMI responsiveness are determined by several cell subsets acting in concert. These results hint at a multifunctional and pluricellular pattern of baseline responsiveness to *S.* Typhi.

## 4 Discussion

Specific immune responses against defined pathogens depend on distinct cell subsets responding to antigenic stimulation (65). Conventional CD4^+^ and CD8^+^ T cells recognize largely bacterial proteins through MHC presentation and generate effector and memory responses, whereas non-conventional populations (MAIT, Tγδ, and NKT) detect different bacterial metabolites through non-classical receptors (e.g. mannose receptor, CD1) (61, 63, 64, 66). For example, CD8^+^ cytotoxic T cells respond rapidly with a clonal expansion and production of IFNγ, TNFα, and granzymes to eliminate infected cells and generate long-lasting memory cells (67). The differentiation of CD4^+^ T cell subsets is contingent on signals (cytokines/chemokines) from APC as the initial responders to defined microorganism. In general, Type 1 helper (Th1) cells produce IFNγ, TNFα, and IL2 in response to intracellular bacteria, Th2 cells promote antibody production against bacterial toxins, Th17 secrete IL17 to protect from extracellular bacteria in mucosal environments, and Th22 promote migration to mucosal surfaces and neutrophil recruitment (65, 67). T_FH_ cells facilitate antibody production and the establishment of B cell memory responses, whilst T_reg_ limit excessive immune activation by downregulating immune responses to contain tissue damage. Ultimately, the efficacy of these protective responses depends on the equilibrium and cooperation among cell subsets, and some patterns might be shared between pathogens; however, the intensity of the inflammation and cell activation depend on the host, the pathogen and their interaction (67).

The composition and functional quality of the T cell compartment before pathogen encounter is increasingly recognized as a determinant of infection outcome; yet for most enteric pathogens, including *S.* Typhi, the baseline cellular landscape remains incompletely defined. For example, in viral infections, the presence of baseline multifunctional CD4^+^ and CD8^+^ T cells, cT_FH_, and Th17 cells has been associated with protection after influenza vaccination, while Tγδ and TNFα-producing CD8^+^ T cells were associated with susceptibility to influenza infection in a generalized logistic regression model (68). This is likely the result, to some extent, of previous exposure to either influenza vaccination and/or infection. Also, baseline T cells showing cross-reactivity to SARS-CoV-2 in uninfected individuals enhanced distinct specific responses to this virus, such as increased frequency of IL2-producing T cells, multifunctional CD4^+^ T cells, and T_FH_ cells, as well as increased CD4^+^ and CD8^+^ T cell proliferation after vaccination (69, 70). The study of baseline T cell responses in SARS-CoV-2 infection is currently being expanded with computational pipelines to identify gene expression signatures (71, 72) to further define the role of baseline response on disease severity and vaccine development (73).

Considerably less information has been reported regarding bacterial infections. Several studies on *S.* Typhi immunity in healthy unexposed individuals have revealed baseline responses to this bacterium in distinct cell subsets, such as monocytes and dendritic cells (24, 28), B cells (31) plasma cells (25), MAIT cells (19, 23), CD8^+^ T memory cells (21, 29), CD4^+^ T regulatory cells (22) and circulating T follicular helper cells (27). In this study, we evaluated the baseline responses to *S.* Typhi in PBMC from 18 healthy unexposed volunteers, using a 52-marker mass cytometry panel (**Supplementary Table 1**). First, we performed supervised (manual) analyses for each cytokine or chemokine, activation, homing, and cytotoxicity marker in CD69^+^ activated cells from each studied T cell subset. Most of these analyses showed a significantly increased *S.* Typhi-specific response from activated T cells when exposed to *S.* Typhi-infected target cells, in comparison to non-infected target cells, pointing to compelling baseline responsiveness to this bacterium in unexposed individuals. We hypothesize that this baseline responsiveness might be due to host cross-reactive responses to other *Enterobacteriacea* present in the gut microbiota. Core members of this family are defined as bacteria with 40-50% homology to *Escherichia coli*, according to phylogenetic studies based on housekeeping genes (74). In silico studies (75, 76) and serologic evaluations (77) have depicted structurally homologous but antigenically diverse epitopes across species, either in structural proteins or in virulence factors. For example, flagellin, a structural protein of bacterial flagella, presents ~50-68% similarity in *E. coli*, *S.* Typhimurium, and *Pseudomonas aeruginosa*, with greater homology (~56-78%) in the binding region and four identical residues in the TLR5 activation site (78). The presence of baseline *S.* Typhi-specific immunity in unexposed individuals might be related to cross-reactive responses to homologous bacterial proteins.

Based on previous data that baseline T cell responses to *S.* Typhi have been associated with protection from disease in wt *S.* Typhi controlled human infection studies (21–23, 27, 29, 47), we aimed to further characterize them in multiple distinct classical and non-conventional T cell subsets and two incubation periods. To this end, we employed a nonlinear dimensionality-reduction technique denominated uniform manifold approximation and projection (UMAP), which has been widely used in mass cytometry datasets, showing high reproducibility and robust cluster organization in considerably low run times (79). Moreover, we combined the clustering ability of the UMAP technique with a high-performance clustering method (PhenoGraph) (80, 81), that conveniently used the same software platform (FlowJo). Data pre-processing, a very important step before analyses (82), was thoroughly performed using quality control methods from the FlowJo software (PeacoQC) and a rigorous cleaning protocol before gating. Clustering quality control was also assessed to avoid over- or underclustering in such a vast dataset.

High-dimensional single-cell data analyses offer distinct advantages to understanding the intricate relationship between different cell subsets in a given setting (82). Unsupervised analyses across all cell subsets from all participants showed a range of 8-61% of validated clusters showing significant increases (p<0.01) and 3-13% of clusters showing significant decreases (p<0.01) when exposed to ST-infected targets compared to NI; only CD8^+^ T_CM_ and T_SCM_ at 18 h did not show significant differences in the generated clusters. Every cell subset behaved differently in each incubation period: various cell subsets showed more significantly different clusters at 40 h than at 18 h (e.g., CD8^+^ T_CM_, CD8^+^ T_EM_, CD4^+^ T_CM_, CD4^+^ T_EMRA_, CD8^+^ T_SCM_, CD8^+^ MAIT, Tγδ), while other subsets showed less clusters at 40 h (e.g., CD8^+^ T_EMRA_, CD4^+^ T_EM_, CD4^+^ T_SCM_, CD4^+^ T_reg_, CD8^+^ T_rp_) and few subsets had similar numbers of significant clusters (e.g., CD4^+^ cT_FH_, NKT). Antigen stimulation time has been reported to affect the functionality or differentiation of the specific CD4^+^ T cell repertoire (37, 38). The differences in behavior related to culture stimulation time likely reflect the dynamic nature of each T cell population over time. Our results support the contention that based on the hypotheses and cell type to be evaluated, selection of a suitable incubation time should be carefully chosen to maximize the interpretation of the results.

As observed in **Table 1** and **Figure 14**, non-conventional T cell subsets, represent some of the subsets with more significant changes in response to ST-infected target cells; NKT cell responses predominate after 18 h incubation (**Table 1** panel A) and Tγδ after 40 h (**Table 1** panel B). These subsets, scarcely described in *S.* Typhi infection, recognize glycolypids and phosphoantigens, respectively, and they have been associated with epigenetic modifications after *S.* Typhi exposure, along with MAIT, monocytes and CD8^+^ T cells (47). In this study, we found highly activated clusters of NKT cells in cultures with *S.* Typhi-infected target cells, both at 18 and 40 h incubation, which aligns with *in vitro* studies showing that CD1d-restricted NKT cells were among the first lymphocytes to be activated after infection of human cells with *S. enterica* serovar Typhimurium (83) and in a mouse model of *S.* Typhimurium infection (84). In these *in vitro* models, the mechanism by which NKT cells respond to *S.* Typhimurium does not depend on bacterial antigens, but on the recognition of host endogenous glycosphingolipids induced by the bacteria (85). The production of IL12 by APC might be another factor inducing NKT responses to *S.* Typhimurium (85), a concern that we addressed in our study by measuring cytokines/chemokines in the cell culture supernatants from the prolonged incubation time (40 h). In the Spearman analyses, we found a positive correlation between NKT cluster 13 and IL12p70 and IL23 (**Supplementary Figure 21**), in agreement with previous reports showing that both cytokines are stimulants for this cell subset in antimicrobial and antitumoral responses (86, 87), which might explain the persistent NKT responses after 40 h incubation. Moreover, these analyses showed a negative correlation between NKT and Tγδ clusters, similar to studies in mice showing an NKT-induced downregulation of IL17-producing Tγδ cells after infection with *S. enterica* serovar Enteritidis. Further studies are needed to elucidate the precise *S.* Typhi antigens inducing human NKT and Tγδ responsiveness, as well as their activation pathways, which might be guided by antigens present on different *Salmonella* serovars which share considerable gene homology (88).

**Figure 14:**
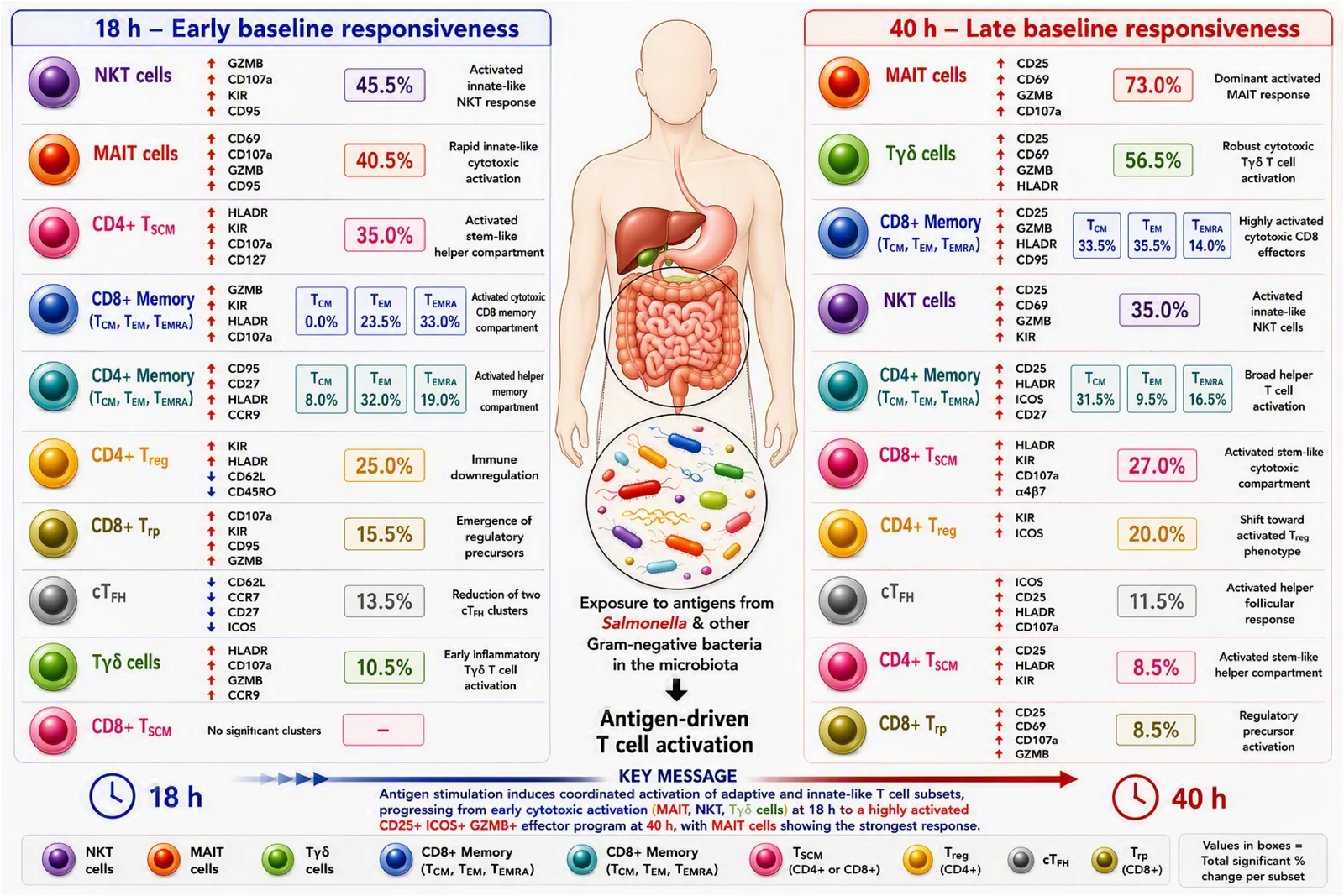
Systemic T cell immunity to *Salmonella enterica* serovar Typhi. Graphical cartoon summary of T cell immune responsiveness observed 18 h and 40 h following exposure to *S.* Typhi infected target cells. Cell subsets are ranked according to the total change per subset. Values in boxes represent the cumulative proportion of clusters that showed significant differences (p<0.0.1) when comparing *S.* Typhi-infected and non-infected target cells. NKT, Natural-Killer T cells. MAIT, CD8^+^ mucosal associated invariant T cells. T_SCM_, T stem cell memory cells. T_CM_, T central memory cells. T_EM_, T effector memory cells. T_EMRA_, T_EM_ re-expressing CD45RA. T_reg_, CD4^+^ T regulatory cells. T_rp_, CD8^+^ T regulatory precursors. cT_FH_, circulating T follicular helper cells. Tγδ, T gamma delta cells.

MAIT cells also represent one of the main subsets involved in the baseline responsiveness to *S.* Typhi at both incubation times, characterized by highly activated cytotoxic clusters with enormous percentage changes in cultures exposed to ST-infected targets compared to NI target cells, as shown in **Table 1** and **Figure 14**. Interestingly, IFNγ-producing MAIT clusters observed after 40 h incubation coincide with results from a previous CHIM study (19) where baseline cytokine-producing MAIT cells (positive to one, two, or three cytokines) were associated with participants that did not develop typhoid fever after exposure to *S.* Typhi (19). The Spearman analyses of 18 h cultures revealed a positive correlation between CCL2 levels with MAIT cluster 10, in accordance with the known CCL2 production by activated MAIT cells (89). Also, IL8 was positively correlated with MAIT cluster 9 in the Spearman analyses in cells collected after 40 h incubation. These results need to be further explored, since IL8 is not traditionally considered a MAIT cell stimulant, but can be produced by this cell subset in response to enterobacterial infections (89).

As expected from our previous studies on CD8^+^ T cells (3, 6, 9, 11–14, 18, 21, 29, 44, 90), where the presence of *S.* Typhi-specific CD8^+^ T cells at baseline is an important immune response associated with protection from disease (21, 29), **Table 1** and **Figure 14** show that these memory subsets were important representatives of baseline responsiveness to *S.* Typhi. For example, we observed that CD8^+^ T_EMRA_, followed by CD8^+^ T_EM_ and CD8^+^ T_EMRA_ at 18 h incubation and CD8^+^ T_EM_ and CD8^+^ T_CM_, followed by CD8^+^ T_EMRA_ at 40 h incubation exhibited pronounced responsiveness. The Spearman analyses with 18 h significant clusters showed a positive correlation of CD8^+^ T_EMRA_ cluster 2 with IL2, which might be related to the intrinsic capacity of CD8^+^ effector cells to secrete IL2 to limit the formation of terminal effector cells during chronic infections (91). However, further studies would be necessary to determine IL2 production in specific CD8^+^ T_EMRA_ cells expressing the markers observed in cluster 2 (e.g. HLADR, CD27, CD95). In the Spearman analyses with cells collected after 40 h incubation, IL33 was positively correlated with CD8^+^ T_EM_ cluster 14, which aligns with the IL33-mediated CD8^+^ T cell expansion shown in mouse models of chronic viral infections (92). These results suggest that the activation of certain cell subsets (e.g., NKT, MAIT, CD8^+^ T_EM_) after 40 h incubation might be enhanced by the presence of cytokines/chemokines within the cell culture supernatants collected ~27 h after initiation of the cultures. Regarding the cytotoxic potential of the CD8^+^ T cell memory subsets, GZMB, a serine protease released from the granules of cytotoxic lymphocytes after antigen exposure, was expressed in CD8^+^ and non-conventional T cell subsets in both incubation periods. Its expression is mediated by activation of TCR and co-stimulatory molecules (93) and mediates cell-death together with perforin in viral and intracellular bacterial infections (94), hence its relevance in evaluating this important mediator in the baseline responsiveness to *S.* Typhi. CD107a was highly expressed in non-conventional T cell subsets and interestingly, in both T_SCM_ subsets. This is also a cytotoxicity marker first described in NK cells and cytotoxic lymphocytes (95). The expression of CD107a in T cells following exposure to ST-infected target cells suggests the development of a cytotoxic response to the intracellular bacteria, but further studies are needed to define the mechanisms related to the interaction of CD107a in different cell types. Moreover, KIR, a member of the human killer-cell immunoglobulin-like receptor (KIR) family, was first described in Natural-Killer (NK) cells (96) and includes inhibitory, activation and regulatory domains that bind to different HLA ligands (97). In this study, the expression of this marker was assessed using a monoclonal antibody that recognizes the extracellular domain of KIR3DL1, which identifies inhibitory KIR^+^ cells. This marker was highly expressed in most cell subsets in both incubation periods, suggesting strong inhibitory signaling after exposure to *S.* Typhi antigens.

Of importance, this study highlights T_SCM_, a novel cell subset that plays a key role in the induction and maintenance of long-term T-CMI (56–58), which has not previously been described in *S.* Typhi immunity. We found *S.* Typhi-specific responses in CD4^+^ T_SCM_ at 18 h and in CD8^+^ T_SCM_ at 40 h, are characterized by the activation marker HLADR and the cytotoxicity marker CD107a, as well as the inhibition marker KIR. These cells expressed several homing markers after 40 h incubation, including the gut homing marker integrin α4β7, suggesting the possible migration of these circulating cell subsets to different organs after exposure to *S.* Typhi. In particular, the CD8^+^ T_SCM_ subset showed more significant clusters after 40 h and no significant correlations with the measured cytokines/chemokines, indicating the necessity of prolonged antigen exposure, probably related to the longevity and plasticity of these cells (52). The T_SCM_ subsets, along with several other conventional and non-conventional T cell subsets, showed activation markers (e.g. HLADR) defining in great depth the characteristics of baseline T-CMI responsiveness to *S.* Typhi. HLADR was one of the most consistent markers highlighted in the unsupervised analyses of all cell subsets, both incubation periods, and present when the validated clusters showed either increases or decreases in co-cultures with ST-infected target cells compared to NI. HLADR is an MHC class II cell surface receptor mainly expressed on activated lymphocytes after re-stimulation of a previously primed T cell population (98). The expression of this marker on monocytes has been used as a predictive or discriminatory biomarker of viral and bacterial infections (99, 100); however, its expression on lymphocytes, either alone or in combination with other markers, such as CD38, has been related to the quantification of the activation response in inflammatory disorders (101, 102). Several studies have investigated a potential anticancer therapeutic strategy using this marker on cytotoxic T lymphocytes, emphasizing its relevance in antitumor T cell responses (103, 104).

CD95 is another activation/exhaustion marker that was expressed as a suggestive baseline feature in most memory T cell subsets and in both incubation periods (**Table 1**). CD95 elicits a death receptor-mediated apoptosis after T cell clonal expansion and effector responses following antigen recognition (105); it facilitates the elimination of antigen-specific cells in the final stages of clonal expansion, where those cells that survive apoptosis remain as a long-term memory subset (106). ICOS is also a key activation marker that was expressed on most conventional T cell subsets and on MAIT cells, particularly after 40 h incubation. Unlike constitutive CD28, ICOS is an inducible marker that supports antigen-specific T cell responses, from activation to differentiation, mainly in effector tissues (107), but its expression after the longer incubation period might be related to cell responses to persistent antigen exposure, as noted in mouse models of chronic viral infections (108). CD25 is the α chain of the IL2 receptor, which is used in the identification of CD4^+^ T regulatory cell subsets, but can also be expressed on T memory cells and other non-immune cells, such as fibroblasts and epithelial cells (109). In this study, CD25 was highly expressed in CD4^+^ T_EM_, CD8^+^ T_rp_, MAIT, and Tγδ, predominantly after 40 h incubation, which suggests this might be a process of immunoregulation in the presence of abundant IL2 produced by other cell types during a prolonged incubation period. Moreover, we observed CD28 expression in CD4^+^ T_EM_ and CD4^+^ cT_FH_, suggesting that this costimulatory molecule associated with the TCR, can also regulate the expression of cytokines/chemokines, particularly IL2 for cell cycle regulation (110, 111). CD27 is a differentiation marker that was consistently expressed on conventional T cell subsets at both incubation periods. CD27 is part of the tumor necrosis factor receptor superfamily that enhances proliferation and persistence of activated T cells, as well as resistance to apoptosis, to fulfill their effector functions, as well as the generation and long-term maintenance of memory (112); its expression suggests that *S.* Typhi-specific T memory cell subsets were activated after exposure to the bacterium. These findings indicate that baseline *S.* Typhi-specific T cell-mediated immunity responsiveness involve predominantly activated cells and are key drivers of successful adaptive responses against pathogens.

Other activation markers, such as CD69, CD39, and CD134, as well as differentiation markers such as CD127 and CD161 were also highlighted in some cell subsets, together with CD71, a proliferation marker, particularly after 40 h incubation. Our results suggested that a prolonged incubation time might stabilize cell cultures to facilitate the activation of some cell subsets that did not thrive after 18 h incubation, such as CD8^+^ T_CM_, CD8^+^ T_EM_, CD4^+^ T_CM_, CD4^+^ T_EMRA_, CD8^+^ T_SCM_, CD8^+^ MAIT, and Tγδ cells. Other subsets such as CD8^+^ T_EMRA_, CD4^+^ T_EM_, CD4^+^ T_SCM_, CD4^+^ T_reg_, and CD8^+^ T_rp_ might not benefit from a prolonged incubation time, since the most notable responses were recorded after 18 h incubation. Interestingly, the Spearman analyses with the 18 h incubation data showed a negative correlation of CD8^+^ T_rp_ cluster 3 with IL17A, in agreement with the opposing functionalities of the proinflammatory IL17A cytokine and a regulatory T cell subset, which has been shown to have an inverse relation to CD8^+^ T regulatory cells in murine models of autoimmune diseases (113). IL33 was also negatively correlated to these regulatory precursors, even though IL33 is known to promote activation of tissue resident CD8^+^ T_reg_ cells in solid tumor environments (114); however, further studies are necessary to determine the precise interaction of this cytokine with the regulatory precursor subset.

Studying the homing potential of circulating *S.* Typhi-specific T cells at baseline could provide additional insights into their significance in the host response to this bacterium. Thus, we studied several homing markers (e.g., CCR7, CD62L, CCR9, integrin α4β7) and whether they were simultaneously expressed in the significant clusters across all cell subsets. Analyses of the expression of these molecules suggested a diverse homing potential of circulating cells to different effector sites as a response to *S.* Typhi antigenic stimulation. These homing markers are extremely relevant in the local responses to this enteric pathogen, either through migration to secondary lymphoid organs (Peyer’s patches), such as CCR7 (115) or CD62L (116), migration to inflamed tissues through CXCR3 (117), or directly to the intestinal mucosa via CCR9 (118) or integrin α4β7 (119). Notably, some of these homing markers were related to the clusters that showed a significant decrease in co-cultures with ST-infected target cells compared to NI. However, our results did not reveal significant clusters of MAIT cells characterized by the expression of homing markers. Of note, a previous report of CCR9 and CCR6 expression on MAIT cells suggested that gut homing of activated MAIT is triggered by exposure to *S.* Typhi in a CHIM study (23). We hypothesize that circulating T cells with diverse homing potential are needed for an effective adaptive response to *S.* Typhi. These cells probably modulate the expression of homing markers just before being recruited to their destination in the intestinal mucosa or lymphoid organs.

Our model mimics *in vivo* antigen presentation using bacteria-infected autologous targets which enables the presentation of diverse bacterial antigens in an autologous system. However, a downside of this approach is that our model cannot pinpoint the precise epitopes driving the T-CMI responses in each cell subset. This will be approached in future studies. Herein, we made every effort to minimize bias using positive and negative experimental controls and the use of a sentinel individual in every experiment. All 18 h and 40 h evaluations were conducted using the same batch of B-LCL and of thawed PBMC. In every experiment, staining cocktails were prepared as a single mix for the staining of both timepoints; 18 h and 40 h samples were run at the Helios™ mass cytometer in consecutive days, always within a 7-day period after staining. Also, as described in the methods and Figures, to avoid potential bias, we performed exhaustive data pre-processing (including quality control and cleaning tools) to mitigate instrument drift. However, a limitation in our study is that we did not control for experimental batch effect in the raw data to avoid over-correction, particularly considering the relatively small size of some of the cell subsets studied. Moreover, intrinsic human variability should also be considered in such a clinical study, where the proportion of each cell subset varied in every participant (see **Table 1**). The overall number of cells evaluated in the mass cytometry experiments restricted the final number of events used to build the concatenated files for the unsupervised analyses, since every volunteer had to be equally represented to avoid bias. Therefore, we emphasize the importance of selecting descriptive statistic tests for non-Gaussian populations and performing paired tests to compare matching data from each volunteer.

In sum, we observed a full breadth of the baseline T cell landscape which includes conventional T cell subsets alongside non-conventional subsets, whose distinct kinetics, antigen-recognition modes, and effector programs may contribute differently to immunity to *S.* Typhi following vaccination and/or infection. T-CMI to *S.* Typhi represents a complex interaction between distinct T cell subsets with multifunctional properties, which we addressed through a high-dimensional data approach. These results enhance previous insights into the baseline cellular responses to *S.* Typhi associated with protection against disease, as well as the previously unknown role of key subsets, such as T_SCM_ and NKT. Of note, the extensive correlation analyses suggest that baseline *S.* Typhi-specific responsiveness is likely the result of multiple subsets acting in concert rather than unique subsets acting independently. Ongoing CHIM studies will help us define the relevance of these findings by studying the associations of these T-CMI baseline responsiveness with clinical outcomes following exposure to wt *S.* Typhi. This information might help guide future investigations of immunity following *S.* Typhi vaccination with live attenuated organisms and/or natural infection.

## Data Availability

The raw data supporting the conclusions of this article will be made available by the authors, without undue reservation.
