# Supplementary material for: Broad systemic T cell immunity to Salmonella enterica serovar Typhi in U.S. healthy adults
Source: Front Immunol. 2026 Oct 1;17:1940148. doi: 10.3389/fimmu.2026.1940148 (PMC13669112; doi:10.3389/fimmu.2026.1940148)
Supplement: Supplementary file 1 [file SupplementaryFile1.docx]

Supplementary Material

# 1 Supplementary Tables

| **Supplementary Table 1. 52 metal-conjugated monoclonal antibody panel for T-CMI** | | | | | | | | |
| --- | --- | --- | --- | --- | --- | --- | --- | --- |
| **#** | **Label** | **Target** | **Clone** | | **Catalog Number** | | **Description / Function** | |
| 1 | 106Cd | CD57 | HNK-1 | | Custom conjugated | | Replicative senescence | |
| 2 | 110Cd | CD56 (NCAM) | NCAM16.2 | | Custom conjugated | | NK & NKT marker; cell adhesion | |
| 3 | 111Cd | CD14 | RMO52 | | Custom conjugated | | Monocyte/macrophage marker; innate LPS recognition | |
| 4 | 111Cd | CD19 | HIB19 | | Custom conjugated | | B cell marker | |
| 5 | 112Cd | CD8a | RPA-T8 | | Custom conjugated | | Cytotoxic T cell marker; MHC I co-receptor | |
| 6 | 113Cd | CD71 | OKT-9 | | Custom conjugated | | Proliferation marker | |
| 7 | 114Cd | CD3 | UCHT1 | | Custom conjugated | | Pan‑T cell marker; TCR signaling | |
| 8 | 115In | CD28 | CD28.2 | | Custom conjugated | | T_SCM_ marker; TCR co-stimulation, lost in terminal effectors | |
| 9 | 116Cd | CD4 | RPA-T4 | | Custom conjugated | | Helper T cell marker; MHC II co‑receptor | |
| 10 | 139La | CD95/Fas | DX2 | | Custom conjugated | | T_SCM_ marker; activation‑induced apoptosis | |
| 11 | 141Pr | CD196 (CCR6) | 11A9 | | 3141014B | | T_h17_ and mucosal homing | |
| 12 | 142Nd | CD134 (OX40) | ACT35 | | Custom conjugated | | Secondary co-stimulatory; immune checkpoint | |
| 13 | 143Nd | CD45RA | HI100 | | 3143006B | | T naïve or T_EMRA_ marker | |
| 14 | 144Nd | Integrin α4β7 | ACT-1 | | Custom conjugated | | Gut mucosal homing | |
| 15 | 145Nd | HLADR | L243 | | Custom conjugated | | T_rp_ marker; sustained activation | |
| 16 | 146Nd | TNFα | Mab11 | | 3146010B | | Inflammatory cytokine | |
| 17 | 147Sm | CD45RO | UCHL1 | | Custom conjugated | | Memory T cell marker | |
| 18 | 148Nd | CD278/ICOS | C398.4A | | 3148019B | | T_FH_ differentiation; immune checkpoint | |
| 19 | 149Sm | CD25 (IL2R) | 2A3 | | 3149010B | | T_reg_ marker; IL2 receptor α chain | |
| 20 | 150Nd | MIP1β | D21-1351 | | 3150004B | | Chemotactic cytokine | |
| 21 | 151Eu | CD107a (LAMP1) | H4A3 | | 3151002B | | Degranulation marker | |
| 22 | 152Sm | TCRγδ | 11F2 | | 3152008B | | γδ T cell receptor | |
| 23 | 153Eu | TCR Vα7.2 | 3C10 | | 3153024B | | MAIT marker; semi-invariant αβ T cell receptor | |
| 24 | 154Sm | CD62L (L-selectin) | DREG-56 | | Custom conjugated | | T_CM_ and T_SCM_ marker; lymph node homing | |
| 25 | 155Gd | CD27 | L128 | | 3155001B | | T_SCM_ and T_rp_ marker; early memory differentiation | |
| 26 | 156Gd | CXCR3 | G025H7 | | 3156004B | | Inflammatory homing | |
| 27 | 158Gd | IL2 | MQ1-17H12 | | 3158007B | | T cell proliferation | |
| 28 | 159Tb | CD197 (CCR7) | G043H7 | | 3159003B | | T_SCM_ marker; lymph node homing | |
| 29 | 160Gd | CD39 | A1 | | 3160004B | | Immunosuppressive ectonucleotidase; exhaustion | |
| 30 | 161Dy | CD152 (CTLA4) | 14D3 | | 3161004B | | Inhibitory checkpoint | |
| 31 | 162Dy | CD69 | FN50 | | 3162001B | | Early activation; lymphocyte retention in lymphoid organs | |
| 32 | 163Dy | CCR9 | L063E8 | | Custom conjugated | | Gut homing | |
| 33 | 164Dy | IL17A | N49-653 | | 3164002B | | T_h17_ effector cytokine | |
| 34 | 165Ho | IFNγ | B27 | | 3165002B | | Type II interferon | |
| 35 | 166Er | IL10 | JES3-9D7 | | 3166008B | | Immune regulation | |
| 36 | 167Er | Granzyme B | GB11 | | Custom conjugated | | Cytotoxic apoptosis | |
| 37 | 168Er | CD154 (CD40L) | 24-31 | | 3168006B | | T‑B cell interaction; activation | |
| 38 | 169Tm | CD366/Tim3 | F38-2E2 | | 3169028B | | Co-inhibitory receptor; late exhaustion | |
| 39 | 170Er | CD122 | Tu27 | | 3170004B | | IL2 receptor β chain; memory support; activation | |
| 40 | 171Yb | CD185/CXCR5 | RF8B2 | | 3171014B | | cT_FH_ marker; follicular homing | |
| 41 | 172Yb | IL21 | 3A3-N2 | | 3172011B | | Lymphoid and myeloid differentiation and function | |
| 42 | 173Yb | CD161 | HP-3G10 | | Custom conjugated | | MAIT marker; C-type lectin receptor | |
| 43 | 174Yb | CD279 (PD1) | EH12.2H7 | | 3174020B | | Immune checkpoint; activation | |
| 44 | 175Lu | CD127 (IL7Ra) | A019D5 | | Custom conjugated | | T_reg_ marker; IL7 receptor α chain | |
| 45 | 176Yb | CD58 (LFA3) | TS2/9 | | 3176017B | | T_SCM_ marker; cell adhesion | |
| 46 | 197Au | KIR | DX9 | | Custom conjugated | | NK inhibition/education | |
| 47 | 209Bi | CD137/4-1BB | 4B4-1 | | 3209015B | | Co-stimulatory immune checkpoint; activation | |
| 48 | 194Pt | Cisplatin | Cell-ID™ | | 201194 | | Viability | |
| 49 | 195Pt | CD45 | HI30 | | 3089003B | | Pan-leukocyte protein | |
| 50 | 196Pt | CD45 | HI30 | | 3195001B | | Pan-leukocyte protein | |
| 51 | 198Pt | CD45 | HI30 | | 3196001B | | Pan-leukocyte protein | |
| 52 | 89Y | CD45 | HI30 | | 3198001B | | Pan-leukocyte protein | |
| Phenotypic markers for cell subsets | | | | | | | | |
| Homing markers | | | |  | |  | |  |
| Activation and Proliferation markers | | | | | | | | |
| Cytokines/chemokines & cytotoxicity | | | | | |  | |  |
| Viability and barcoding | | | |  | |  | |  |
| NK, Natural Killer cells. NKT, Natural-Killer T cells. LPS, lipopolysaccharide. MHC, Major Histocompatibility Complex. TCR, T cell receptor. T_SCM_, T stem cell memory. T_h17_, T helper 17 cells. T_EMRA_, T effector memory re-expressing CD45RA cells. T_rp_, T regulatory precursor. T_FH_, T follicular helper cells. T_reg_, T regulatory cells. MAIT, mucosal-associated invariant T cells. T_CM_, T central memory cells. cT_FH_, circulating T follicular helper cells. | | | | | | | | |

# 2 Supplementary Figures

**Supplementary Figure 1. Peak Extraction and Cleaning Oriented Quality Control (PeacoQC) process results.** FCS files were imported into FlowJo Software (version 10.10.0, BD) for quality control through the PeacoQC plugin (version 1.5). The figure shows an example of the peak detection (pink lines) using an input isolation tree (IT) parameter of 0.80, and outlier removal (purple areas) using an input mean absolute deviation (MAD) of 8.00. Both colors represent the areas removed from each FCS file before further analysis (“Bad events”). The PeacoQC “Good Events” were exported into a new FlowJo workspace after these analyses to continue the analytical strategy shown in **Supplementary Figure 2**.

**Supplementary Figure 2. Analytical Strategy.** PeacoQC “Good events” were further analyzed through sequential gatings to remove doublets, debris and calibration beads, to generate a “clean up” file for downstream analyses. After gating on viable cells, each FCS file was debarcoded into the original 6 culture samples, which were exported to a new Flow Jo workspace as individual FCS files for analyses.

**Supplementary Figure 3. Gating strategies for the different T cell subsets evaluated in this study**. **Panel A** shows the gating strategy for T_CM_, T central memory cells, T_EM_, T effector memory cells, T_EMRA_, T effector memory cells re-expressing CD45RA, and T_SCM_, T stem memory cells. **Panel B** shows the gating strategy for cT_FH_, circulating T follicular helper cells, T_reg_, T regulatory cells, T_rp_, T regulatory precursors, MAIT, mucosal-associated invariant T cells, Tγδ, gamma-delta T cells, and NKT, Natural Killer T cells.

**Supplementary Figure 4. Unsupervised analyses workflow.** Step-by-step description of the unsupervised analyses performed separately for each studied cell subset after 18 h and 40 h incubation.

**Supplementary Figure 5.** ***Salmonella*-specific cytokine production (IFNγ and TNFα), gut homing marker expression (α4β7 integrin and CCR9), and/or cytotoxicity marker expression (CD107a and granzyme B) in CD8^+^ T cell subsets after ex-vivo exposure to *S.* Typhi-infected autologous targets in 18 healthy volunteers**. Supervised analysis of baseline PBMC responses after an 18 h co-culture with autologous EBV-transformed lymphoblastoid B cell lines showing the differences between non-infected (NI, blue) and *S.* Typhi-infected (ST, red) targets. Comparisons were evaluated with Wilcoxon match-pairs signed rank tests, using GraphPad software version 11.0.0. Medians with interquartile ranges are shown in each bar, each dot represents individual data from each participant. T_CM_, T central memory cells. T_EM_, T effector memory cells. T_EMRA_, T effector memory cells re-expressing CD45RA. T_SCM_, T stem memory cells. T_rp_, T regulatory precursors. ns, not significant. * p<0.05, ** p<0.01, *** p<0.001, **** p<0.0001.

**Supplementary Figure 6.** ***Salmonella*-specific cytokine production (IFNγ and TNFα), homing marker expression (integrin α4β7, CCR9, CXCR3, and CCR7), and/or cytotoxicity marker expression (CD107a) in CD4^+^ T cell subsets after ex-vivo exposure to *S.* Typhi-infected autologous targets in 18 healthy volunteers**. Supervised analysis of baseline PBMC responses after an 18 h co-culture with autologous EBV-transformed lymphoblastoid B cell lines showing the differences between non-infected (NI, blue) and *S.* Typhi-infected (ST, red) targets. Comparisons were evaluated with Wilcoxon match-pairs signed rank tests, using GraphPad software version 11.0.0. Medians with interquartile ranges are shown in each bar, each dot represents individual data from each participant. T_CM_, T central memory cells. T_EM_, T effector memory cells. T_EMRA_, T effector memory cells re-expressing CD45RA. T_SCM_, T stem memory cells. T_reg_, T regulatory cells. cT_FH_, circulating T follicular helper cells. * p<0.05, ** p<0.01, *** p<0.001, **** p<0.0001.

**Supplementary Figure 7.** ***Salmonella*-specific cytokine production (TNFα), gut homing marker expression (integrin α4β7), and cytotoxicity marker expression (CD107a and granzyme B) in non-conventional T cell subsets after ex-vivo exposure to *S.* Typhi-infected autologous targets in 18 healthy volunteers**. Supervised analysis of baseline PBMC responses after an 18 h co-culture with autologous EBV-transformed lymphoblastoid B cell lines showing the differences between non-infected (NI, blue) and *S.* Typhi-infected (ST, red) targets. Comparisons were evaluated with Wilcoxon match-pairs signed rank tests, using GraphPad software version 11.0.0. Medians with interquartile ranges are shown in each bar, each dot represents individual data from each participant. MAIT, mucosal-associated invariant T cells. Tγδ, gamma-delta T cells. NKT, Natural Killer T cells. ns, not significant. * p<0.05, *** p<0.001, **** p<0.0001.

**Supplementary Figure 8. Supervised validation of hierarchical clusterings of concatenated CD8^+^ T central memory (T_CM_) cells after ex-vivo exposure to *S.* Typhi-infected autologous targets in 18 healthy volunteers.** Supervised validation of PhenoGraph clusters visualized on UMAP reference maps of CD8^+^ T_CM_ cells after a 40 h PBMC co-culture with autologous EBV-transformed lymphoblastoid B cell lines showing significant increases (p<0.01) between non-infected (NI, blue) and *S.* Typhi (ST, red)-infected targets. Comparisons were performed with Wilcoxon match-pairs signed rank tests, using GraphPad software version 11.0.0. The corresponding percentage change (gray) was calculated using a conventional formula: $\left[ \frac{ST-NI}{NI} \right]*100$. Medians with interquartile ranges are shown in each bar, each dot represents individual data from each participant; markers selected for the supervised gating to validate each cluster are shown below each graph. *** p<0.001, **** p<0.0001.

**Supplementary Figure 9. Supervised validation of hierarchical clusterings of concatenated CD8^+^ T effector memory (T_EM_) cells after ex-vivo exposure to *S.* Typhi-infected autologous targets in 18 healthy volunteers.** Supervised validation of PhenoGraph clusters visualized on UMAP reference maps of CD8^+^ T_EM_ cells after an 18 h **(A)** and 40 h **(B)** PBMC co-culture with autologous EBV-transformed lymphoblastoid B cell lines showing significant differences (p<0.01) between non-infected (NI, blue) and *S.* Typhi (ST, red)-infected targets. Comparisons were performed with Wilcoxon match-pairs signed rank tests, using GraphPad software version 11.0.0. The corresponding percentage change (gray) was calculated using a conventional formula: $\left[ \frac{ST-NI}{NI} \right]*100$. Medians with interquartile ranges are shown in each bar, each dot represents individual data from each participant; markers selected for the supervised gating to validate each cluster are shown below each graph. *** p<0.001, **** p<0.0001.

**Supplementary Figure 10. Supervised validation of hierarchical clusterings of concatenated CD8^+^ T effector memory cells re-expressing CD45RA (T_EMRA_) after ex-vivo exposure to *S.* Typhi-infected autologous targets in 18 healthy volunteers.** Supervised validation of PhenoGraph clusters visualized on UMAP reference maps of CD8^+^ T_EMRA_ cells after an 18 h **(A)** and 40 h **(B)** PBMC co-culture with autologous EBV-transformed lymphoblastoid B cell lines showing significant increases (p<0.01) between non-infected (NI, blue) and *S.* Typhi (ST, red)-infected targets. Comparisons were performed with Wilcoxon match-pairs signed rank tests, using GraphPad software version 11.0.0. The corresponding percentage change (gray) was calculated using a conventional formula: $\left[ \frac{ST-NI}{NI} \right]*100$. Medians with interquartile ranges are shown in each bar, each dot represents individual data from each participant; markers selected for the supervised gating to validate each cluster are shown below each graph. ** p<0.01, *** p<0.001.

**Supplementary Figure 11. Supervised validation of hierarchical clusterings of concatenated CD4^+^ T central memory (T_CM_) cells after ex-vivo exposure to *S.* Typhi-infected autologous targets in 18 healthy volunteers.** Supervised validation of PhenoGraph clusters visualized on UMAP reference maps of CD4^+^ T_CM_ cells after an 18 h **(A)** and 40 h **(B)** PBMC co-culture with autologous EBV-transformed lymphoblastoid B cell lines showing significant increases (p<0.01) between non-infected (NI, blue) and *S.* Typhi (ST, red)-infected targets. Comparisons were performed with Wilcoxon match-pairs signed rank tests, using GraphPad software version 11.0.0. The corresponding percentage change (gray) was calculated using a conventional formula: $\left[ \frac{ST-NI}{NI} \right]*100$. Medians with interquartile ranges are shown in each bar, each dot represents individual data from each participant; markers selected for the supervised gating to validate each cluster are shown below each graph. ** p<0.01, *** p<0.001, **** p<0.0001.

**Supplementary Figure 12. Supervised validation of hierarchical clusterings of concatenated CD4^+^ T effector memory (T_EM_) cells after ex-vivo exposure to *S.* Typhi-infected autologous targets in 18 healthy volunteers.** Supervised validation of PhenoGraph clusters visualized on UMAP reference maps of CD4^+^ T_EM_ cells after an 18 h **(A)** and 40 h **(B)** PBMC co-culture with autologous EBV-transformed lymphoblastoid B cell lines showing significant increases (p<0.01) between non-infected (NI, blue) and *S.* Typhi (ST, red)-infected targets. Comparisons were performed with Wilcoxon match-pairs signed rank tests, using GraphPad software version 11.0.0. The corresponding percentage change (gray) was calculated using a conventional formula: $\left[ \frac{ST-NI}{NI} \right]*100$. Medians with interquartile ranges are shown in each bar, each dot represents individual data from each participant; markers selected for the supervised gating to validate each cluster are shown below each graph. ** p<0.01, *** p<0.001.

**Supplementary Figure 13. Supervised validation of hierarchical clusterings of concatenated CD4^+^ T stem cell memory (T_SCM_) cells after ex-vivo exposure to *S.* Typhi-infected autologous targets in 18 healthy volunteers.** PhenoGraph clusters visualized on a UMAP reference map of CD4^+^ T_SCM_ cells after a 40 h **(A)** PBMC co-culture with autologous EBV-transformed lymphoblastoid B cell lines. Panel **B** shows hierarchical clustering of concatenated CD4^+^ T_SCM_ cells after 40 h incubation. The arrow shows a cluster with significant increase (p<0.01) after comparing non-infected (NI) and *S.* Typhi (ST)-infected targets. The percentage shown within the graph is the estimated representation of the changing cluster. A heatmap was generated after clustering of concatenated CD4^+^ T_SCM_ cells after 40 h **(C)** incubation. The cluster with a significant increase is denoted by a black outlined rectangle. The varying levels of intensity on the expression of each marker are shown in the blue (low) to red (high) scale. Panels **D** and **E** depict the validation of the clusters showing significant differences between NI (blue) and ST (red) target cells, after 40 h and 18 h incubation, respectively. The corresponding percentage change (gray) was calculated using a conventional formula: $\left[ \frac{ST-NI}{NI} \right]*100$. Comparisons between groups were evaluated with Wilcoxon match-pairs signed rank tests, using GraphPad software version 11.0.0. Medians with interquartile ranges are shown in each bar, each dot represents individual data from each participant (panels **D** and **E**); markers selected for the supervised gating to validate the unsupervised analysis are shown below each graph. ** p<0.01, *** p<0.001, **** p<0.0001.

**Supplementary Figure 14. Supervised validation of hierarchical clusterings of concatenated CD8^+^ T stem cell memory (T_SCM_) cells after ex-vivo exposure to *S.* Typhi-infected autologous targets in 18 healthy volunteers.** PhenoGraph clusters visualized on a UMAP reference map of CD8^+^ T_SCM_ cells after an 18 h **(A)** PBMC co-culture with autologous EBV-transformed lymphoblastoid B cell lines. Panel **B** shows hierarchical clustering of concatenated CD8^+^ T_SCM_ cells after 18 h incubation. A heatmap was generated after clustering of concatenated CD8^+^ T_SCM_ cells after 18 h **(C)** incubation. The varying levels of intensity on the expression of each marker are shown in the blue (low) to red (high) scale. Panel **D** depict the validation of the additional clusters showing significant differences between NI (blue) and ST (red) target cells, after 40 h incubation, according to the heatmap in **Figure 9**. The corresponding percentage change (gray) was calculated using a conventional formula: $\left[ \frac{ST-NI}{NI} \right]*100$. Comparisons between groups were evaluated with Wilcoxon match-pairs signed rank tests, using GraphPad software version 11.0.0. Medians with interquartile ranges are shown in each bar, each dot represents individual data from each participant (panel **D**); markers selected for the supervised gating to validate the unsupervised analysis are shown below each graph. ** p<0.01, *** p<0.001.

**Supplementary Figure 15. Supervised validation of hierarchical clusterings of concatenated CD4^+^ T regulatory (T_reg_) cells after ex-vivo exposure to *S.* Typhi-infected autologous targets in 18 healthy volunteers.** A heatmap was generated after clustering of concatenated CD4^+^ T_reg_ cells after 18 h **(A)** and 40 h **(C)** incubation. The varying levels of intensity on the expression of each marker are shown in the blue (low) to red (high) scale. Panels **B** and **D** depict the validation of the additional clusters showing significant differences between NI (blue) and ST (red) target cells. The corresponding percentage change (gray) was calculated using a conventional formula: $\left[ \frac{ST-NI}{NI} \right]*100$. Comparisons between groups were evaluated with Wilcoxon match-pairs signed rank tests, using GraphPad software version 11.0.0. Medians with interquartile ranges are shown in each bar, each dot represents individual data from each participant (panels **B** and **D**); markers selected for the supervised gating to validate the unsupervised analysis are shown below each graph. ** p<0.01, *** p<0.001.

**Supplementary Figure 16. Supervised validation of hierarchical clusterings of concatenated CD8^+^ T regulatory precursors (T_rp_) cells after ex-vivo exposure to *S.* Typhi-infected autologous targets in 18 healthy volunteers.** A heatmap was generated after clustering of concatenated CD8^+^ T_rp_ cells after 18 h **(A)** and 40 h **(C)** incubation. The varying levels of intensity on the expression of each marker are shown in the blue (low) to red (high) scale. Panel **B** depicts the validation of the additional cluster showing a significant increase between NI (blue) and ST (red) target cells. The corresponding percentage change (gray) was calculated using a conventional formula: $\left[ \frac{ST-NI}{NI} \right]*100$. Comparisons between groups were evaluated with Wilcoxon match-pairs signed rank tests, using GraphPad software version 11.0.0. Medians with interquartile ranges are shown in each bar, each dot represents individual data from each participant (panel **B**); markers selected for the supervised gating to validate the unsupervised analysis are shown below each graph. ** p<0.01.

**Supplementary Figure 17. Supervised validation of hierarchical clusterings of concatenated CD8^+^ mucosal associated invariant T (MAIT) cells after ex-vivo exposure to *S.* Typhi-infected autologous targets in 18 healthy volunteers.** Supervised validation of PhenoGraph clusters visualized on UMAP reference maps of CD8^+^ MAIT cells after an 18 h **(A)** and 40 h **(B)** PBMC co-culture with autologous EBV-transformed lymphoblastoid B cell lines showing significant differences (p<0.01) between non-infected (NI, blue) and *S.* Typhi (ST, red)-infected targets. Comparisons were performed with Wilcoxon match-pairs signed rank tests, using GraphPad software version 11.0.0. The corresponding percentage change (gray) was calculated using a conventional formula: $\left[ \frac{ST-NI}{NI} \right]*100$. Medians with interquartile ranges are shown in each bar, each dot represents individual data from each participant; markers selected for the supervised gating to validate each cluster are shown below each graph. ** p<0.01, *** p<0.001, **** p<0.0001.

**Supplementary Figure 18. Supervised validation of hierarchical clusterings of concatenated T gamma delta (Tγδ) cells after ex-vivo exposure to *S.* Typhi-infected autologous targets in 18 healthy volunteers.** Supervised validation of PhenoGraph clusters visualized on UMAP reference maps of Tγδ cells after an 18 h **(A)** and 40 h **(B)** PBMC co-culture with autologous EBV-transformed lymphoblastoid B cell lines showing significant increases (p<0.01) between non-infected (NI, blue) and *S.* Typhi (ST, red)-infected targets. Comparisons were performed with Wilcoxon match-pairs signed rank tests, using GraphPad software version 11.0.0. The corresponding percentage change (gray) was calculated using a conventional formula: $\left[ \frac{ST-NI}{NI} \right]*100$. Medians with interquartile ranges are shown in each bar, each dot represents individual data from each participant; markers selected for the supervised gating to validate each cluster are shown below each graph. ** p<0.01, *** p<0.001, **** p<0.0001.

**Supplementary Figure 19. Supervised validation of hierarchical clusterings of concatenated Natural Killer T (NKT) cells after ex-vivo exposure to *S.* Typhi-infected autologous targets in 18 healthy volunteers.** Supervised validation of PhenoGraph clusters visualized on UMAP reference maps of NKT cells after an 18 h **(A)** and 40 h **(B)** PBMC co-culture with autologous EBV-transformed lymphoblastoid B cell lines showing significant increases (p<0.01) between non-infected (NI, blue) and *S.* Typhi (ST, red)-infected targets. Comparisons were performed with Wilcoxon match-pairs signed rank tests, using GraphPad software version 11.0.0. The corresponding percentage change (gray) was calculated using a conventional formula: $\left[ \frac{ST-NI}{NI} \right]*100$. Medians with interquartile ranges are shown in each bar, each dot represents individual data from each participant; markers selected for the supervised gating to validate each cluster are shown below each graph. ** p<0.01, *** p<0.001, **** p<0.0001.

**Supplementary Figure 20. Correlation between cytokines measured in cell culture supernatants and clusters showing significant differences in the various cell subsets identified by unsupervised analyses, according to cell culture incubation periods.** Clusters with significant differences (p ≤ 0.01) between non-infected (NI) and S. Typhi (ST)-infected targets after 18 h (A, n=39) or 40 h (B, n=55) PBMC co-culture with autologous B-LCL were selected to perform a non-parametric Spearman correlation analysis. Cytokines measured in cell culture supernatants (n=10) collected at 27 h (the time at which the monensin/brefeldin A blockers were added to the 40 h cell cultures) were also included in the Spearman’s analyses. Fields with significant differences (* p ≤ 0.01) are colored according to their Spearman correlation coefficient in a blue (1.0) to red (-1.0) scale. To enhance readability, only data showing significant p values are depicted in the heatmaps. Labels indicate the cell subsets (e.g., CD4TSCM) followed by the cluster number showing a significant difference.

**Supplementary Figure 21. Correlation between cytokines measured in cell culture supernatants and clusters showing significant differences by unsupervised analyses at 18 and 40 h, classified by cell subsets.** After validation of cell subsets by unsupervised analyses, conventional T cells (A: CD4+, B: CD8+) or non-conventional T cells (C: MAIT, NKT, and Tγδ) clusters with significant differences (p ≤ 0.01) between non-infected (NI) and S. Typhi (ST)-infected targets after an 18 h or 40 h PBMC co-culture with autologous B-LCL were selected to perform non-parametric Spearman correlation analyses. Cytokines measured in cell culture supernatants (n=10) collected at 27 h (the time at which the monensin/brefeldin A blockers were added to the 40 h cell cultures) were also included in the Spearman’s analyses. Fields with significant differences (* p ≤ 0.01) are colored according to their Spearman correlation coefficient in a blue (1.0) to red (-1.0) scale. To enhance readability, only data showing significant p values are depicted in the heatmaps. Labels indicate the cell subsets (e.g., CD4TSCM) followed by the cluster number showing a significant difference.

**Supplementary Figure 22. Spearman correlation analyses between cytokines measured in cell culture supernatants and clusters showing significant differences in the various cell subsets identified by unsupervised analyses.** Clusters with significant differences (p ≤ 0.01) between non-infected (NI) and *S.* Typhi (ST)-infected targets after 18 h (n=39) or 40 h (n=55) PBMC co-culture with autologous B-LCL were selected to perform a non-parametric Spearman correlation analysis. Cytokines measured in cell culture supernatants (n=10) collected at 27 h (the time at which the monensin/brefeldin A blockers were added to the 40 h cell cultures) were also included in the Spearman’s analyses. Fields are colored according to their Spearman correlation coefficient in a blue (1.0) to red (-1.0) scale. Labels indicate the cell subsets (e.g., CD4TSCM) followed by the cluster number.
